# Systematic review and meta analysis of mechanical properties of 3D printed denture bases compared to milled and conventional materials

**DOI:** 10.1038/s41598-025-14288-2

**Published:** 2025-08-09

**Authors:** Amr Azab, Walid Awad Abdelhady, Enas Elwakeel, Mohamed Ashraf, Rim Wally, Amir Soliman, Maged Ahmed Mohamed, Dina Abozaid

**Affiliations:** 1 Prosthodontics Department, Faculty of Dentistry, Tanta University, Tanta, Egypt; 2 Crown and Bridge Department, Faculty of Dental Medicine, Al-Azhar University, Cairo, Egypt; 3 Faculty of Dentistry, Tanta University, Tanta, Egypt; 4 Faculty of Dental Medicine, Al-Azhar University, Cairo, Egypt; 5 Faculty of Dentistry, Suez Canal University, Ismailia, Egypt; 6 Faculty of Oral and Dental Medicine, Future University in Egypt, Cairo, Egypt; 7 Medical Research Group of Egypt, NEGIDA ACADEMY LLC , Massachusetts Avenue, Arlington, MA 02474 USA; 8 Dental Biomaterials Department, Faculty of Dentistry, Tanta University, Tanta, Egypt

**Keywords:** 3D printing, CAD-CAM milling, Denture base materials, Flextural Strength, Surface hardness, Systematic review, meta-analysis, Health care, Dentistry, Dental treatments, Prosthetic dentistry

## Abstract

Denture base fabrication has advanced with the introduction of computer-aided design and manufacturing (CAD-CAM) techniques, such as subtractive milling and additive 3D printing. However, concerns persist regarding the mechanical performance of 3D-printed denture bases. This systematic review and meta-analysis aimed to evaluate and compare the flexural strength (FS), surface hardness, fracture toughness, and impact strength of 3D-printed denture bases with those produced by milling and conventional methods. A systematic search of PubMed, Scopus, Web of Science, and Cochrane Central was conducted up to March 2025 in accordance with Preferred Reporting Items for Systematic Reviews and Meta-Analyses (PRISMA) guidelines. In vitro studies comparing 3D-printed denture bases with milled or conventional heat-polymerized bases in terms of mechanical properties were included. The Joanna Briggs Institute (JBI) checklist for quasi-experimental studies was used. Data was extracted, and quantitative synthesis was performed where possible. Thirty-eight studies were included, comprising 562 specimens for FS and 231 for surface hardness. Meta-analysis revealed that milled denture bases demonstrated the highest flexural strength (MD = -1.11, 95% CI [-1.29, -0.93], *p* < 0.001) and surface hardness (MD = -26.49, 95% CI [-29.89, -23.10], *p* < 0.001) compared to 3D-printed bases. Conventional bases outperformed 3D-printed ones in most mechanical properties. Milled denture bases exhibited the highest FS (120–146 MPa), followed by conventional PMMA (95–119 MPa), while 3D-printed bases showed wider variability (28–128 MPa). Surface hardness (VHN), fracture toughness (MPa·m¹/²), and impact strength (kJ/m²) were also superior in milled bases. Statistical heterogeneity was present due to differences in materials, printing orientation, and post-curing protocols. Subgroup analysis based on printing orientation (0°, 45°, and 90°) partially explained this variability, showing higher FS in horizontally printed specimens. Although 3D-printed denture bases offer customization and production efficiency, their mechanical properties remain inferior to milled alternatives. Optimization of resin formulations, printing parameters, and post-processing protocols is essential to enhance their clinical performance. The main limitations were high heterogeneity among included studies, differences in material formulations, variability in testing standards, and the in vitro nature of most included studies. This review was registered in PROSPERO (CRD420250639092). There were no deviations from the registered protocol.

**Supplementary Information:**

The online version contains supplementary material available at 10.1038/s41598-025-14288-2.

## Introduction

Complete denture fabrication is very important management for edentulism^1^. It has undergone significant advancements since the introduction of polymethylmethacrylate (PMMA) in the 1930s. PMMA remains the most widely used material for denture base resins because of its high mechanical strength, dimensional stability, biocompatibility, and ease of processing^2,3^. Despite these advantages, conventional denture bases are susceptible to midline fractures, mechanical fatigue, and polymerization shrinkage, which can compromise clinical longevity^4,5^. Notably, up to 63% of dentures may fracture within the first three years of use, emphasizing the need for improved materials and fabrication techniques^6,7^.

To address the limitations of conventional compression-molded dentures, alternative methods such as chemically activated resins, thermoplastic injection molding, and light-activated resins were introduced. While these innovations improved strength and reduced polymerization shrinkage, they also posed challenges such as residual monomer content, limited adaptation, and occasional allergic reactions^8–10^.

In recent years, the advent of computer-aided design and manufacturing (CAD-CAM) technologies has revolutionized denture base fabrication. CAD-CAM techniques are classified into subtractive (milling) and additive (3D printing) methods^1^. Subtractive CAD-CAM milling fabricates denture bases by cutting pre-polymerized PMMA blocks using computer-controlled milling machines, producing dense, homogeneous structures. Additive 3D printing builds denture bases layer by layer from liquid resin using technologies such as SLA or DLP, followed by post-curing to achieve final properties.

Milled denture bases, created from pre-polymerized resin blocks, exhibit superior mechanical properties, including higher fracture toughness, reduced porosity, and better dimensional accuracy compared to conventional methods^11,12^ . Milled denture base materials typically exhibit higher mechanical properties than 3D-printed ones due to the use of highly cross-linked, pre-polymerized PMMA blocks, which result in reduced porosity, enhanced polymerization, and greater homogeneity. In contrast, 3D-printed materials may suffer from incomplete polymerization, interlayer defects, and orientation-dependent weaknesses. However, milling is material-intensive and generates significant waste, which has spurred interest in additive manufacturing as a more sustainable alternative^13,14^.

3D printing technologies, such as stereolithography (SLA), fused deposition modeling (FDM), and selective laser sintering (SLS), fabricate dentures layer by layer, allowing for precise, complex designs with minimal material wastage. This method also reduces production time and costs, making it an attractive option for both clinicians and patients^4,15^. Nevertheless, 3D-printed dentures often have lower mechanical properties, including FS and hardness, compared to milled counterparts^3,16,17^.

The mechanical properties of denture bases, particularly flexural strength, surface hardness, fracture toughness, and impact resistance, are critical for clinical performance and patient safety. Dentures must resist masticatory forces, thermal cycling, and wear over time, and poor mechanical properties can lead to fracture, deformation, and biofilm retention. Dentures are subjected to repeated thermal cycling, which can degrade material properties over time^4,16^.

Studies have shown that CAD-CAM milled resins typically outperform 3D-printed materials in thermal stress resistance^18,19^. Also, surface properties such as roughness, microhardness, and color stability are crucial determinants of durability and patient satisfaction. For instance, rougher surfaces can promote microbial adhesion, leading to biofilm formation and staining, while smoother, harder surfaces resist wear and microbial colonization^10,20,21^.

The choice of fabrication method also influences denture hygiene and maintenance. Chemical denture cleansers, commonly used to maintain oral health, can affect the surface properties of denture bases. Frequent exposure to these cleansers may alter the mechanical properties of the material, including surface roughness and hardness, impacting long-term performance^11,22^. Despite the growing adoption of digital fabrication methods, there remains a lack of comprehensive data comparing the mechanical properties of 3D-printed, milled, and conventional denture base materials. Key properties such as FS, fracture toughness, and resistance to thermal and mechanical stresses must meet clinical standards to ensure success^23,24^.

Given the inherent differences in material structure and fabrication protocols, it is expected that milled, 3D-printed, and conventionally processed bases will exhibit distinct mechanical behaviors. For example, milling uses highly cross-linked PMMA blocks with uniform polymerization, whereas 3D printing is affected by build angle, resin formulation, and post-curing techniques. Several studies have compared these fabrication methods using in vitro testing, yet no prior systematic review has comprehensively synthesized their mechanical properties across multiple outcomes. Moreover, the influence of key factors such as printing orientation, post-processing protocols, and material type remains unclear.

Therefore, this systematic review and meta-analysis aims to evaluate and compare the flexural strength, surface hardness, fracture toughness, and impact strength of 3D-printed denture base materials against milled and conventional alternatives. It further seeks to identify processing variables that influence mechanical performance, ultimately guiding clinical decision-making for durable and efficient prosthodontic rehabilitation.

## Materials and methods

### Study design & search strategy

**Fig. 1:**
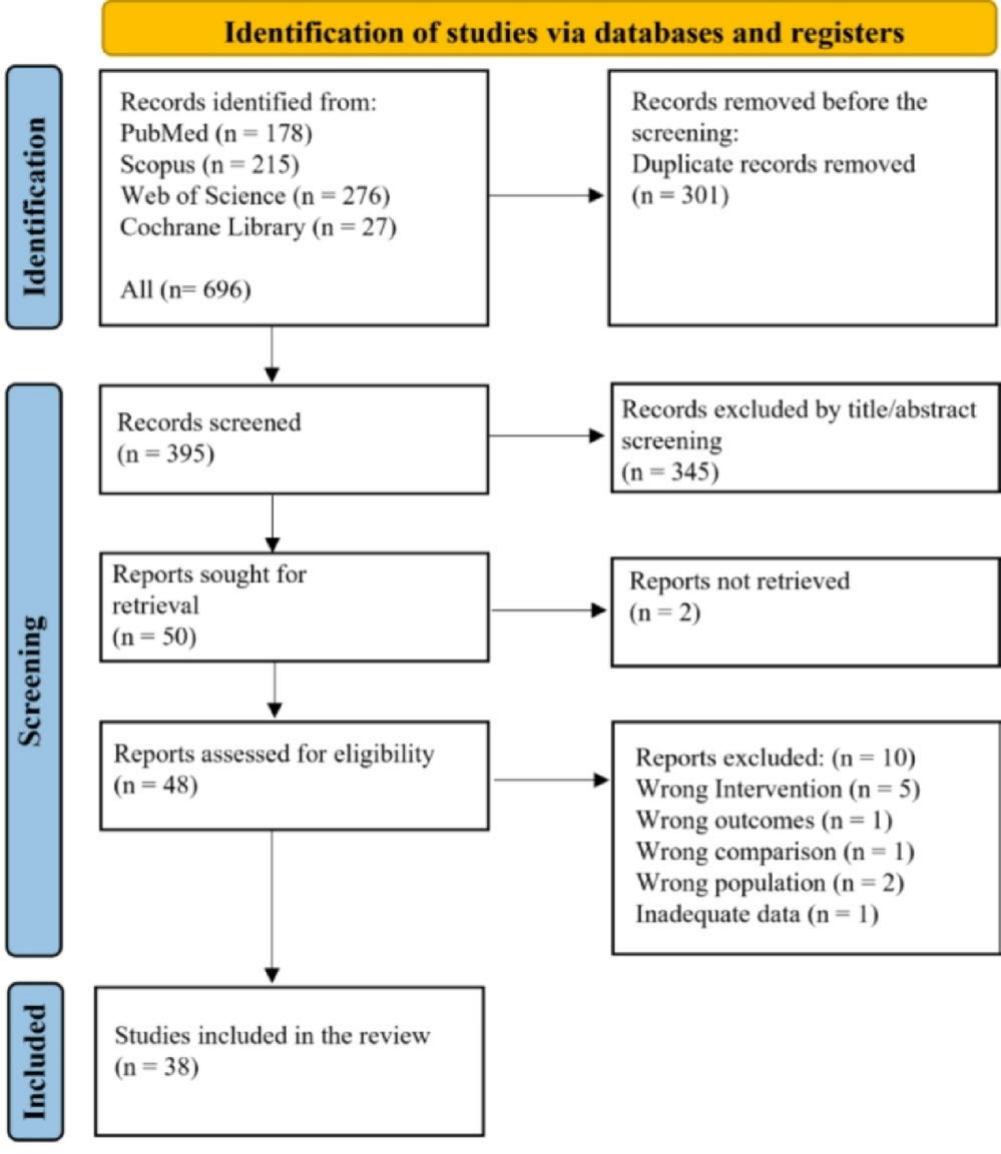
PRISMA flow chart.

This systematic review was conducted in accordance with the Preferred Reporting Items for Systematic Reviews and Meta-Analyses (PRISMA) guidelines (Fig. 1).

A comprehensive literature search was performed in December 2024 across four major electronic databases: PubMed, Scopus, Web of Science, and the Cochrane Central Register of Controlled Trials. The search strategy included a combination of keywords related to denture base fabrication and material properties, such as “3D printing,” “CAD-CAM milling,” “denture base,” “mechanical properties,” “additive manufacturing,” and “subtractive manufacturing.” The complete search strings and Boolean operators for each database are provided in Supplementary Table S1.

To ensure a systematic and unbiased screening process, all identified records were imported into Rayyan – Intelligent Systematic Review (Rayyan QCRI, Free Web Version), for both title/abstract screening and full-text review. Rayyan (Qatar Computing Research Institute, Doha, Qatar) is a web-based tool designed to facilitate collaborative and blinded screening by multiple reviewers, with features for resolving conflicts and tracking inclusion/exclusion decisions^25^. This approach enabled efficient management of search results and consistent application of the inclusion and exclusion criteria throughout the study selection phase. Additional variables extracted included resin brand, build orientation, post-curing protocol, country of study, and thermocycling conditions.

For comparability, only studies using standardized specimen geometries and testing protocols were included in the quantitative synthesis. Where differences existed, data were normalized or analyzed separately in subgroup analyses. When details were missing and could not be retrieved from the study, those data points were excluded from the quantitative synthesis to maintain accuracy and transparency in the analysis. No assumptions were made.

### Study selection criteria

Studies were included if they compared mechanical properties of 3D-printed denture bases with milled or conventional heat-polymerized bases, regardless of resin brand, printing technology (e.g., SLA, DLP, FDM), or post-processing protocol. Studies were excluded if they used only aesthetic or color stability outcomes, or if they were case reports, reviews, or non-experimental studies.

**Inclusion criteria**.

Studies were included if:

- Were in vitro experimental studies.
- Compared 3D-printed denture base materials to milled or conventional heat-polymerized PMMA bases.
- Reported at least one mechanical property: flexural strength, surface hardness, fracture toughness, or impact strength.
- Used standardized specimen geometry (e.g., ISO 20795-1).
- Provided quantitative outcomes.
- Were published in English with full-text available.

**Exclusion criteria**:

Studies were excluded if:

- Focused solely on aesthetic or color outcomes.
- Were case reports, reviews, or editorials.
- Employed unconventional specimen shapes or unclear fabrication/testing protocols.

### Data extraction and quality assessment

Data were independently extracted by two reviewers using a pre-designed data extraction form. Extracted data included study design, country, material type, fabrication technique, printer model, layer thickness, build orientation, post-curing protocol, mechanical test method, and outcome values. A detailed summary of each included study’s resin type, printing orientation, layer thickness, post-curing protocol, and mechanical testing method is provided in Supplementary Table S3. Each step (screening, data extraction and quality assessment) was performed independently by two authors, and a third author reviewed all data for consistency and accuracy.

### Quality assessment method

The Joanna Briggs Institute (JBI) Checklist for Quasi-Experimental Studies was used to assess risk of bias across nine domains, focusing on:

- Selection bias.
- Confounding factors.
- Intervention validity.
- Outcome measurement reliability.
- Statistical validity.

Disagreements between reviewers during study selection or risk of bias assessment were resolved through discussion and consensus, with a third reviewer consulted if necessary. Also, results were compared to ISO 20795-1 and ADA Specification No. 12 benchmarks for denture base materials where applicable.

### Meta analysis assessment

Meta-analyses were performed using Review Manager (RevMan) version 5.4. Continuous outcomes were synthesized using inverse-variance Fixed-effects models and reported as mean differences (MD) with 95% confidence intervals (CI). Heterogeneity was assessed using the I² statistic, with I² values > 50% considered substantial. The subgroup analyses explored potential sources of heterogeneity, including material type, post-processing techniques, and printing orientation. Specifically, studies were stratified by build angle (0°, 45°, 90°) where data permitted, to evaluate the influence of printing direction on mechanical performance.

Studies were grouped for meta-analysis based on resin type, printer type, and build orientation. Sensitivity analyses excluded high-Sensitivity analyses excluded high-risk-of-bias studies to assess robustness. Publication bias was evaluated via funnel plots if ≥ 10 studies were included.

## Results

### Study characteristics

The systematic search yielded 696 records. After removing duplicates, 395 unique articles remained for title and abstract screening. Of these, 345 articles were excluded for irrelevance or failure to meet the inclusion criteria. Forty-eight articles were deemed potentially eligible based on preliminary screening. After a full-text review, a total of 38 in vitro studies were included in this review (Table 1). The number of studies contributing data for each mechanical property was:

Flexural strength (FS): 21 studies.

Surface hardness: 19 studies.

Fracture toughness: 5 studies.

Impact strength: 4 studies.

The distribution of studies by country showed that the highest number were conducted in Saudi Arabia (6 studies) and Egypt (5 studies), followed by Brazil and the USA (4 studies each) (Table 1). India contributed 3 studies, while Italy, Turkey, and Croatia each contributed 2 studies. Other countries, including Romania, Korea, Switzerland, Canada, Jordan, Portugal, and Taiwan, each contributed 1 study, highlighting a broad international research landscape.

**Table 1:** Summary of included Studies.

Study	Country	Related Measured Outcomes	Significance
^26^	Egypt	Flexural Strength	CAD-CAM milled denture base resins (DBRs) exhibited superior flexural strength compared to conventional compression-molded or 3D-printed DBRs, whereas 3D-printed DBRs and polyamide demonstrated the lowest flexural strengths.
^27^	Egypt	Surface Roughness, Microhardness	Chemical denture cleansers (CDCs) significantly influenced the surface properties of denture base materials (DBMs), with Corega showing the most adverse effects on roughness and color stability, and H2O2 markedly reducing microhardness. Prolonged use of CDCs warrants caution.
^16^	Egypt	Vickers Hardness, Surface Roughness, Fracture Toughness	Milled specimens displayed lower surface roughness and higher hardness and fracture toughness than 3D-printed specimens, both before and after thermocycling. Thermocycling reduced hardness and fracture toughness while increasing surface roughness in both groups, though it had no impact on water sorption or solubility.
^28^	India	Fracture Toughness	Formlabs and Dentca (3D-printed) exhibited significantly lower fracture toughness than Leucitone 199 (conventional), while Leucitone 199 was inferior to Avadent (CAD-CAM) in fracture toughness.
^29^	UK	Flexural strength, Impact strength, and hardness	Fused deposition modeling (FDM) samples, though cost-effective and reproducible, revealed that current testing standards for conventional denture polymers are unsuitable for additive-manufactured materials, necessitating new protocols for clinical implementation.
^30^	Saudi Arabia	Flexural Strength and Elastic Modulus	Heat-polymerized, AvaDent, and IvoCad materials are viable for denture bases at 1.5 mm thickness, whereas FormLabs and NextDent require a minimum thickness of 2 mm to achieve clinically acceptable flexural properties.
^31^	Saudi Arabia	Flexural Strength and Flexural Modulus	Milled groups outperformed printed groups in flexural strength and modulus but were more susceptible to aging and cyclic loading. AvaDent showed the highest flexural strength in controls, Dentsply Block in fatigued groups, and Dentca, Dentsply Block, and Keystone in thermocycled groups. Lucitone 3D exhibited the highest flexural strength when repaired with composite.
^32^	India	Compressive Strength, Flexural Strength, and Hardness	Milled PMMA and thermopressing demonstrated superior compressive strength, while thermopressing also excelled in flexural strength and hardness. The 3D-printed resin exhibited the lowest color stability.
^33^	Brazil	Candidal adhesion and their effects on the surface, optical, and mechanical properties like surface microhardness, flexural strength, and modulus of elasticity	Despite the growing use of CAD/CAM-fabricated dentures, limited research exists on how denture cleaners affect microbial adhesion and material properties, highlighting the need for further investigation.
^34^	Romania	Tensile strength, Vickers Hardness	Heat-cured resins remain clinically acceptable due to their surface quality, mechanical properties, and affordability. CAD/CAM milled resins showed the best mechanical properties and surface finishes, while 3D printing is suitable for provisional solutions.
^35^	Brazil	Knoop Microhardness, Flexural Strength, and Modulus of Elasticity	CAD/CAM-milled resins matched traditional resins in mechanical properties, whereas 3D-printed resins were inadequate for long-term use, though ongoing research aims to improve their performance.
^36^	USA	Flexural Strength, Fracture Toughness	3D-printed denture base materials exhibited mechanical, optical, and physical properties comparable to conventional and milled materials.
^37^	Turkey	Vickers Microhardness and Flexural Strength	Graphene-reinforced PMMA showed the highest flexural strength and microhardness, unaffected by thermal cycling, which otherwise reduced these properties in other resins.
^38^	Italy	Flexural Strength	Polymerization techniques significantly influenced flexural strength in acrylic and composite resins. Temp Print, combined with pink resin powder, emerged as a promising alternative for removable dentures.
^39^	USA	Fracture Toughness	Milled samples with embedded 3D-printed titanium frameworks exhibited higher impact resistance, flexural strength, and lower elastic deformation compared to non-framework milled or printed samples.
^40^	Italy	Ultimate Flexural Strength, Flexural Strain, Flexural Modulus	CAD-milled PMMA displayed optimal flexural properties, lowest pre-polishing roughness, and reduced bacterial adhesion after 90 min, though all materials showed similar roughness and microbial adhesion after 16 h.
^41^	Egypt	Impact Strength, Flexural Strength, and Surface Roughness	Milled specimens had higher flexural and impact strength and lower roughness than 3D-printed specimens. Polishing reduced roughness in printed specimens but had no significant effect on milled ones.
^42^	Brazil	Flexure Strength	CAD-CAM milled PMMA showed the lowest *C. albicans* biofilm formation and highest flexural strength, while 3D-printed specimens had the lowest strength and highest roughness.
^43^	Saudi Arabia	Flexural strength, Impact strength, and Surface hardness	3D-printed resin had inferior flexural strength, impact strength, and hardness compared to heat-polymerized resin but superior surface roughness. Thermal cycling reduced hardness and flexural strength while increasing roughness, with no effect on impact strength.
^44^	Egypt	Surface Hardness, Fracture Toughness	Milled specimens exhibited higher hardness and fracture toughness than 3D-printed specimens before and after immersion in denture cleansers, which reduced these properties in both groups without affecting water sorption or solubility.
^7^	Korea	Flexural strength and modulus	3D-printed materials demonstrated suitable mechanical properties for hard dental prostheses.
^45^	Croatia	Flexural Strength, Surface Hardness	Digital denture manufacturing techniques influence residual monomer content, flexural strength, and microhardness, though these factors alone do not guarantee optimal properties.
^46^	USA	Flexural Strength, Flexural Strain	All digitally fabricated denture base materials met clinical acceptability standards, even after hard relining, though flexural strength varied by material type.
^47^	USA	Flexural Strength	3D-printed denture base materials exhibited flexural strength comparable to or lower than milled materials, with thermal cycling further reducing their strength.
^2^	Saudi Arabia	Flexural strength, Elastic modulus, and Surface Hardness	CAD-CAM milled resins surpassed heat-polymerized and 3D-printed resins in flexural strength, modulus, and hardness, though 3D-printed resins remained clinically acceptable.
^2^	Saudi Arabia	Surface hardness	Denture base resin properties varied, with CAD/CAM and thermoformed resins maintaining hardness and color stability after brushing, though surface roughness was affected.
^48^	Switzerland	Flexural Strength and Fracture Toughness	CAD-CAM milled and 3D-printed denture resins showed similar biocompatibility and roughness, but milled resins were mechanically superior. Printing orientation and printer type influenced resin strength and roughness.
^8^	Egypt	Surface Hardness and Impact Strength	CAD/CAM milled resins had the lowest surface roughness and highest impact strength and hardness compared to 3D-printed and polyamide resins.
^49^	Saudi Arabia	Surface hardness	Denture base material and disinfectants affected surface properties, with lasers producing smoother surfaces and improving CAD/CAM resin hardness, while chemical disinfectants enhanced PMMA hardness.
^4^	Jordan	Surface hardness, Flexural properties, and Impact strength	3D-printed resins exhibited variations in surface and mechanical properties compared to conventional PMMA, necessitating further research before standardization.
^13^	Saudi Arabia	Surface Roughness, Flexure strain, Maximum load, Flexure stress at yield, and Flexure modulus	CAD-CAM resins outperformed 3D-printed resins in surface and mechanical properties, with build plate angles having no significant effect on 3D-printed resin roughness.
^50^	Portugal	Microhardness and Flexural strength	Printed resins had lower microhardness than conventional resins but comparable flexural strength.
^32^	India	Vickers hardness and Color stability	Milled PMMA showed superior color stability, while tooth-shade resins (milled and 3D-printed) exhibited higher hardness than pink-shade resins. Tooth-shade 3D-printed resin also outperformed pink-shade in color stability and hardness.
^51^	Brazil	Flexural strength and Elastic modulus	3D-printed resin demonstrated fatigue strength and surface roughness comparable to subtractive and pressed methods, supporting its potential for dental prostheses.
^52^	Turkey	Flexural strength	Digitally produced denture bases exhibited higher flexural strength than conventionally manufactured bases.
^3^	Croatia	Flexural strength and Surface hardness	CAD/CAM materials had the highest surface hardness, while 3D-printed materials showed the lowest flexural strength.
^53^	Turkey	Shear bond strength	Sandblasting was most effective for shear bond strength (SBS) in conventional resins, while laser treatment worked best for additive-manufactured resins. Subtractive resins showed similar SBS across surface treatments except plasma.
^54^	Germany	Maximum fracture forces	The testing setup effectively evaluated denture fracture behavior, demonstrating that digital design and manufacturing can enhance mechanical stability, particularly with optimized dentition forms.

The synthesis of mechanical properties across the included studies encompassed a diverse range of denture base materials fabricated via conventional heat-polymerization, CAD/CAM milling, and various 3D printing technologies (Table 1). The studies varied in sample sizes, resin brands, and testing protocols, yet collectively provided comprehensive data on fracture toughness, FS, hardness, and related mechanical outcomes. Most studies employed standardized in vitro testing methods, ensuring comparability of results. For each included study, detailed information on key mechanical testing protocols, including specimen dimensions, loading rates, post-curing protocols, resin brand and type, build orientation, and thermocycling conditions, was extracted and summarized. This comprehensive summary is presented in Supplementary (Table S3**)** to facilitate comparison and interpretation of results across studies. Units used for testing each property are:

- MPa (megapascal) for flexural strength.
- VHN (Vickers Hardness Number) for surface hardness.
- (megapascal times square root of meter) MPa·m¹/² for fracture toughness.
- (kilojoule per square meter) kJ/m² for impact strength.

For comparison between values of surface hardness Knoop hardness number was converted to Vicker hardness number for standardization.

### Quality assessment results

The *JBI* checklist showed a low risk of bias in all studies for questions 1, 2, 3, 4, 6,7 and 9. Question 5 showed low risk in 18 studies, high risk in 5 studies and was not applicable to 16 studies. Question 8 was not applicable to all studies included (Table 2).

**Table 2:** *JBI* checklist of included studies.

No.	Study	Q1	Q2	Q3	Q4	Q5	Q6	Q7	Q8	Q9
1	^26^	Yes	Yes	Yes	Yes	N/A	Yes	Yes	N/A	Yes
2	^27^	Yes	Yes	Yes	Yes	N/A	Yes	Yes	N/A	Yes
3	^16^	Yes	Yes	Yes	Yes	N/A	Yes	Yes	N/A	Yes
4	^28^	Yes	Yes	Yes	Yes	N/A	Yes	Yes	N/A	Yes
5	^29^	Yes	Yes	Yes	Yes	N/A	Yes	Yes	N/A	Yes
6	^30^	Yes	Yes	Yes	Yes	N/A	Yes	Yes	N/A	Yes
7	^31^	Yes	Yes	Yes	Yes	N/A	Yes	Yes	N/A	Yes
8	^32^	Yes	Yes	Yes	Yes	N/A	Yes	Yes	N/A	Yes
9	^33^	Yes	Yes	Yes	Yes	N/A	Yes	Yes	N/A	Yes
10	^34^	Yes	Yes	Yes	Yes	N/A	Yes	Yes	N/A	Yes
11	^35^	Yes	Yes	Yes	Yes	N/A	Yes	Yes	N/A	Yes
12	^36^	Yes	Yes	Yes	Yes	N/A	Yes	Yes	N/A	Yes
13	^37^	Yes	Yes	Yes	Yes	N/A	Yes	Yes	N/A	Yes
14	^38^	Yes	Yes	Yes	Yes	N/A	Yes		N/A	Yes
15	^39^	Yes	Yes	Yes	Yes	N/A	Yes	Yes	N/A	Yes
16	^40^	Yes	Yes	Yes	Yes	N/A	Yes	Yes	N/A	Yes
17	^41^	Yes	Yes	Yes	Yes	N/A	Yes	Yes	N/A	Yes
18	^42^	Yes	Yes	Yes	Yes	N/A	Yes	Yes	N/A	Yes
19	^43^	Yes	Yes	Yes	Yes	N/A	Yes	Yes	N/A	Yes
20	^44^	Yes	Yes	Yes	Yes	N/A	Yes	Yes	N/A	Yes
21	^7^	Yes	Yes	Yes	Yes	N/A	Yes	Yes	N/A	Yes
22	^45^	Yes	Yes	Yes	Yes	N/A	Yes	Yes	N/A	Yes
23	^46^	Yes	Yes	Yes	Yes	N/A	Yes	Yes	N/A	Yes
24	^47^	Yes	Yes	Yes	Yes	N/A	Yes	Yes	N/A	Yes
25	^2^	Yes	Yes	Yes	Yes	N/A	Yes	Yes	N/A	Yes
26	^2^	Yes	Yes	Yes	Yes	N/A	Yes	Yes	N/A	Yes
27	^48^	Yes	Yes	Yes	Yes	N/A	Yes	Yes	N/A	Yes
28	^8^	Yes	Yes	Yes	Yes	N/A	Yes	Yes	N/A	Yes
29	^49^	Yes	Yes	Yes	Yes	N/A	Yes	Yes	N/A	Yes
30	^4^	Yes	Yes	Yes	Yes	N/A	Yes	Yes	N/A	Yes
31	^13^	Yes	Yes	Yes	Yes	N/A	Yes	Yes	N/A	Yes
32	^50^	Yes	Yes	Yes	Yes	N/A	Yes	Yes	N/A	Yes
33	^32^	Yes	Yes	Yes	Yes	N/A	Yes	Yes	N/A	Yes
34	^51^	Yes	Yes	Yes	Yes	N/A	Yes	Yes	N/A	Yes
35	^52^	Yes	Yes	Yes	Yes	N/A	Yes	Yes	N/A	Yes
36	^3^	Yes	Yes	Yes	Yes	N/A	Yes	Yes	N/A	Yes
37	^53^	Yes	Yes	Yes	Yes	N/A	Yes	Yes	N/A	Yes
38	^54^	Yes	No	Yes	Yes	N/A	Yes	Yes	N/A	Yes

Risk of bias assessments indicated that the majority of included studies demonstrated low to moderate risk, with common limitations related to incomplete reporting of randomization and blinding procedures. Overall, the quality of evidence was sufficient to support meaningful synthesis, though some variability in methodological rigor was noted and considered in the interpretation of findings.

### Meta analysis results

#### The meta-analysis of flexural strength

Meta-analysis showed that 3D-printed denture bases had significantly lower flexural strength compared to both milled and conventional bases.

- 3D-printed vs. conventional: Mean difference (MD) = − 11.93 MPa, 95% CI [− 12.51, − 11.36], *p* < 0.001.
- 3D-printed vs. milled: MD = − 1.11 MPa, 95% CI [− 1.29, − 0.93], *p* < 0.001.

**Fig. 2:**
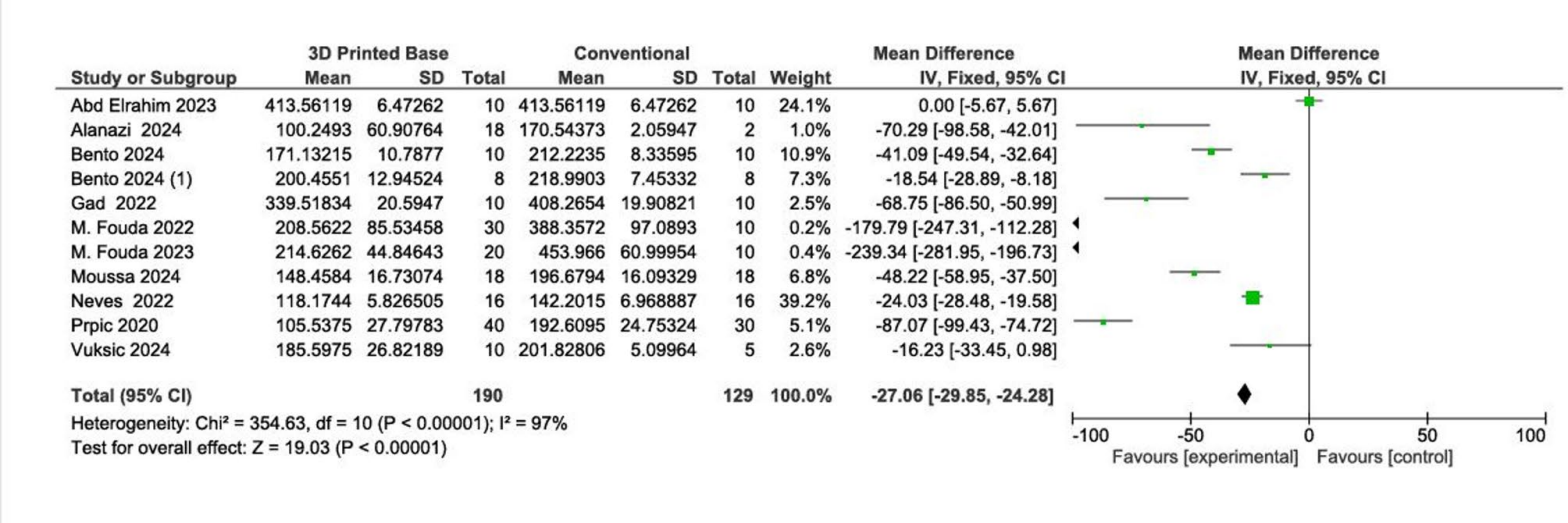
Pooled results for flexural strength (3d printed vs. conventional denture base).

**Fig. 3:**
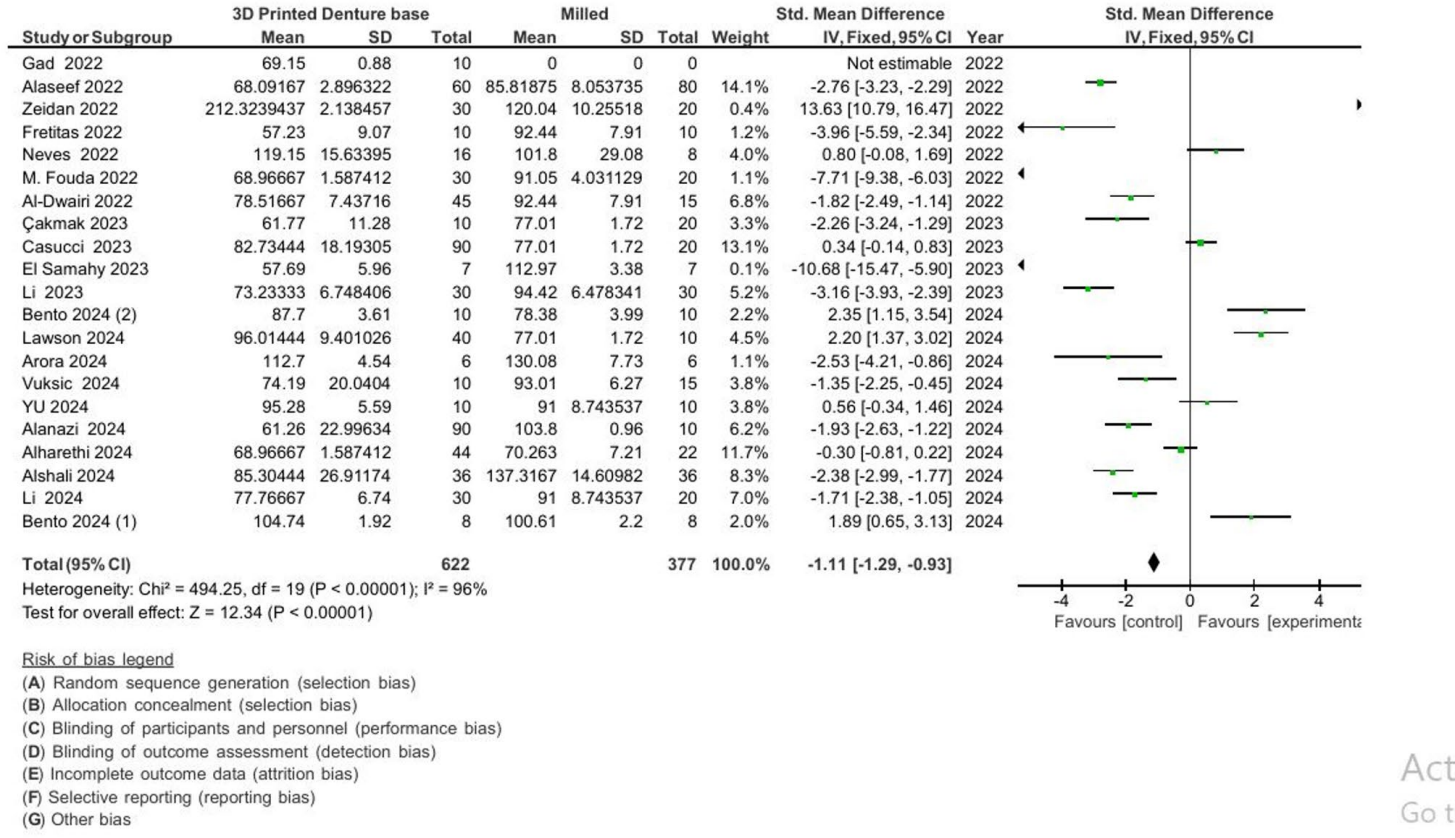
Pooled results for flexural strength (3d printed vs. milled denture base).

The Pooled results for flexural strength are represented in Figs. 2 and 3. The GRADE summary of findings for the mechanical properties of 3D-printed, milled, and conventional denture base materials is presented in Supplementary Table S2.

#### The meta-analysis of hardness

These findings indicate that milled denture bases had the highest flexural strength and surface hardness.

- 3D-printed vs. conventional: MD = − 27.06 VHN, 95% CI [− 29.85, − 24.28], *p* < 0.001.
- 3D-printed vs. milled: MD = − 26.49 VHN, 95% CI [− 29.89, − 23.10], *p* < 0.001.

**Fig. 4:**
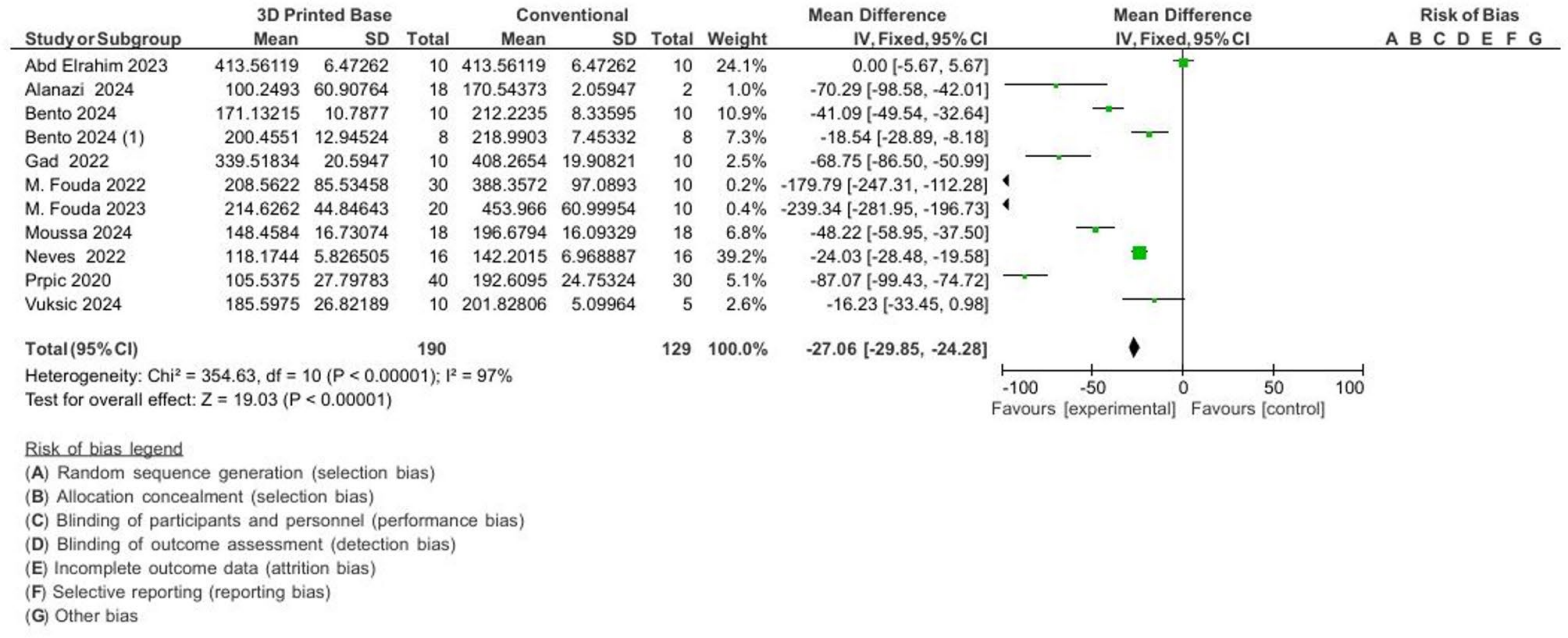
Pooled results for hardness (3d printed vs. conventional denture base).

**Fig. 5:**
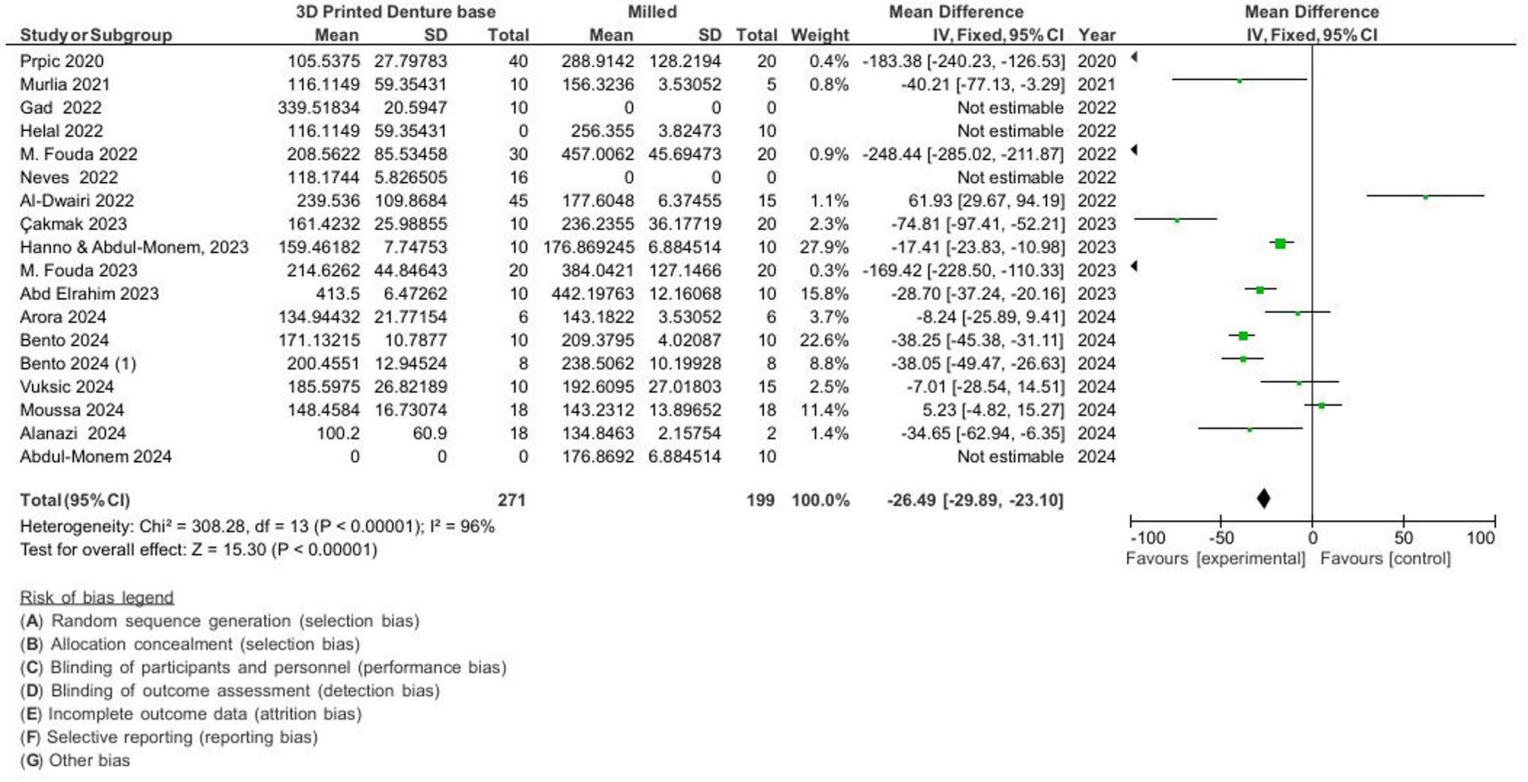
Pooled results for hardness (3d printed vs. milled denture base).

The Pooled results For hardness measurements are presented in Figs. 4 and 5.

#### Heterogeneity and publication bias

Substantial heterogeneity was observed:

- Flexural strength: I² = 78%, *p* < 0.001.
- Surface hardness: I² = 73%, *p* < 0.001.

Funnel plots were generated for FS and surface hardness (outcomes with ≥ 10 studies). Slight asymmetry suggested possible publication bias, but trim-and-fill analysis indicated only minimal impact on pooled effect sizes (adjustment < 5%).

#### Subgroup and sensitivity analyses

- Subgroup analysis by printing orientation (0°, 45°, 90°) showed that horizontally printed (0°) specimens had significantly higher flexural strength.
- Sensitivity analysis excluding high-risk-of-bias studies confirmed the robustness of the findings, with no change in the direction or statistical significance of results.

### Flexural strength (FS)

Twenty-one studies evaluated the flexural strength of 3D-printed denture base materials compared to other manufacturing methods. The findings of these studies are summarized in Table 3.

**Table 3:** Flexural strength values and study settings for included studies.

Study	Groups	Resin brand	Printing parameters			Flexural Strength (dry) (MPa) Mean ± SD	
			Layer thickness	Building angle	Printing technique	Mean	SD
^26^	Heat-polymerized	Vertex	100 μm	45°		99	16.53
	Milled DBRs	AvaDent				120.49	13.07
	Milled DBRs	Polident				119.59	6.25
	3D-printed DBRs	Harz			DLP	28.82	1.94
	3D-printed DBRs	NextDent				61.63	2.32
	Polyamide	AvaDent				33.28	0.79
^29^	aHeat cured	ProBase^®^ Hot (Ivoclar Vivadent AG)				91.94	8
	Cold cured	ProBase^®^ Cold (Ivoclar Vivadent AG)				117.25	1.92
	Milled	Ivoclar Vivadent AG				103.8	0.96
	3D-printed FDM	Material4print		x	FDM	65.35	10.25
	3D-printed FDM			y	FDM	34.92	8.33
	3D-printed FDM			z	FDM	23.39	0.68
	3D-printed SLA	Formlabs Inc.		x	SLA	53.5	0.96
	3D-printed SLA			y	SLA	61.83	0.64
	3D-printed SLA			z	SLA	61.19	2.24
	3D-printed SLA			x	SLA	89.38	8.65
	3D-printed SLA			y	SLA	70.48	4.81
	3D-printed SLA			z	SLA	91.3	7.37
^30^	Heatpolymerized	Major.Base.20		90°		86.86	3.1
	Milled	AvaDent,				93.95	2.9
	Milled	Ivo CAD				87.05	1.5
	3Dprinted	FormLabs			SLA	70.14	2.8
	3Dprinted	NextDent			DLP	68.24	1.3
	Heatpolymerized	Major.Base.20				82.07	2.7
	Milled	AvaDent,				92.43	2.6
	Milled	Ivo CAD				83.59	3.7
	3Dprinted	FormLabs			SLA	69.82	3.8
	3Dprinted	NextDent			DLP	66.7	1.9
	Heat polymerized	Major.Base.20				72.67	1.7
	Milled	AvaDent,				92.87	1.6
	Milled	Ivo CAD				82.15	2.8
	3Dprinted	FormLabs			SLA	67.39	2.4
	3Dprinted	NextDent			DLP	66.26	1.6
	Heat polymerized	Major.Base.20				67.4	2.2
	Milled	AvaDent,				84.09	2.6
	Milled	Ivo CAD				70.42	2.1
	3Dprinted	FormLabs			SLA		
	3Dprinted	NextDent			DLP		
^31^	3Dprinted	Lucitone	50 μm	90°		128.55	10.3
	3Dprinted	Formlabs				122.52	13.66
	3Dprinted	Dentca				108.12	2.17
	Milled	AvaDent				146	12.53
	Milled	Lucitone 199				125.51	14.84
	Milled	Keystone				140.44	4.26
^32^	3Dprinted	Asiga	100 μm	horizontal orientation		112.7	4.54
	Milled	Ivotion				130.08	7.73
	Thermopressing	Polyan IC				152.96	27
	Injection molding	SR-Ivocap				131.28	22.21
^33^	Heatpolymerized	Classic resin	50 μm	45°		104.19	2.47
	Milled	BlueDent				100.61	2.2
	3Dprinted	SmartDent				104.74	1.92
^35^	Conventional	(medium pink color, Onda Cryl, Clássico)				73.12	4.57
	Microwave	(medium pink color, Onda Cryl, Clássico)				76.41	6.01
	Milled	(medium pink color, BlueDent, Articon)				78.38	3.99
	3D-printed	SmartDent			SLA + DLP	87.7	3.61
^36^	Milled	Ivotion	100 μm	horizontal		77.01	1.72
	Heat polymerized	Lucitone 199				96.36	2.43
	3D-printed vat DLP	Denture Base II			DLP	97.03	4.87
	3D-printed vat DLP	High Impact Denture Base			DLP	102.4	3.15
	3D-printed vat CLIP	Lucitone Digital Print			CLIP	82.36	1.68
	3D printed vat Material jetting	TrueDent Resin			Material jetting	101.76	7.65
^37^	Milled	G-CAM	?	horizontal		95.95	10.8
	Milled	Ivotion				86.66	6.03
	3D-printed resin	Denturetec			DLP	61.77	11.28
^38^	Heatpolymerized	Ruthinium-Dental	?	0°		89.15	14.31
	Cold-Cured	Ruthinium-Dental				86.07	7.09
	Cold-Cured	Ruthinium-Dental				74.83	7.84
	Heatpolymerized	Ruthinium-Dental				85.58	8.6
	Heatpolymerized	Ruthinium-Dental				92.39	17.18
	Heatpolymerized	Ruthinium-Dental				98.86	10.66
	Milled	Ivoclar vivadent, Schaan, Liechtenstein				91.88	4.43
	Milled	GC				107.87	7.56
	3D-printed PMMA	NextDent			DLP	60.11	5.72
	3D-printed PMMA	SprintRay			DLP	54.07	3.55
	3D-printed PMMA	NextDent			DLP	83.32	8.38
	3D-printed PMMA	SprintRay			DLP	85.44	5.3
^41^	Milling	(M-PM; Merz Dental GmbH)				112.97	3.38
	3d printing	Formlabs				57.69	5.96
^42^	CAD-CAM	AvaDent	50	90		114.96	16.23
	3D printing	Cosmos				57.23	9.07
	microwave	Vipi Wave				108.09	11.75
	conventional heat polymerizing	Lucitone 199				108.94	9.14
^43^	heat polymerizing	Major.Base.20	50	90		86.63	1
	3D-printed	(NextDent)				69.15	0.88
^7^	3d printing	Ivocap				95.28	5.59
	milling	Vipi Block Gum				93.01	6.27
	conventional	THD				119.11	9.29
^45^	heat cure	Meliodent				97.06	6.25
	Thermosens	Vertex				62.57	5.69
	CAD pink V	Ivobase				79.06	4.65
	CAD pink	Polident				96.27	5.81
	CAD pink U	Anaxdent				83.31	3.21
	CAD-CAM denture base material,	Freeprint denture				103.33	16.71
	CAD-CAM denture base material, additive manufacturing	Imprimo LC denture				69.75	7.63
	Lucitone 199	Dentsply Sirona				66.27	4.02
^46^	IvoBase CAD	Ivoclar AG				89.59	0.82
	Dentsply Sirona	Lucitone 199				102.3	1.03
	Argen DS HI-Base	Argen Corp				91.37	4.35
	Detax Freeprint Denture	Detax GmbH & Co				68.48	5.17
	Asiga DentaBase	Asiga				70.83	3.96
	Dreve Fotodent Denture	Dreve Dentamid GmbH				80.42	1.38
^47^	Lucitone 199	Lucitone 199				73.8	4.4
	Lucitone Digital Fit	Lucitone Digital Fit				98.9	3.5
	Ivotion Base	Ivotion Base				83.1	1.4
	Lucitone Digital Print	Lucitone Digital Print				83.6	2.9
	Flexcera Base	Flexcera Base				78.9	1.6
	FotoDent Dentures	FotoDent Dentures				70.8	5.7
^2^	Heat-polymerized	Major.Base.20,	50	90		82.7	1.6
	AvaDent	AvaDent denture base puck				94.5	2.2
	IvoCad	IvoBase CAD				87.6	1.1
	ASIGA	ASIGA			DLP	70.1	0.8
	FormLabs	FormLabs			SLA	69.1	1.5
	NextDent	Denture 3D+			SLA	67.7	1.2
^4^	M (meliodent)	NextDent, Dentona and ASIGA				92.44	7.91
	3D Dentona	Dentona	50 μm			81.33	5.88
	3D Asiga	ASIGA				79.33	6.07
	3D Next Dent	Next Dent				74.89	8.44
^13^	3D printed 120	ASIGA	50 μm	120°	DLP	35.881	3.72
	3D printed 135			135°	DLP	42.734	4.44
	Milled	IvoBase				70.263	7.21
^50^	3D printed	V-Print	50 μm			123.8	18.33
	3D printed	NextDent				114.5	10.17
	Heat-cured	Probase Hot				107.7	27.7
	Heat-cured	Villacryl Rapid				101.8	29.08

#### 3D-printed vs. milled materials

When comparing 3D-printed materials milled ones regarding FS, results varied among studies. However, most studies reported that CAD-CAM milled denture bases generally exhibited superior FS^2,26,31,34,40,41^. For instance, milled *AvaDent* and *Keystone* materials demonstrated values exceeding 140 megapascal (MPa), while the highest-performing 3D-printed material *Dentca* reached 128.55 ± 10.30 MPa^31^. Milled materials proved superior to 3D-printed ones again both before and after thermocycling, with a statistically significant difference (*p < 0.05*)^41^.

On the other hand, some unusually reported that the 3D-printed materials exhibited better flexural properties compared to the milled ones^7,35,36,45,52^. The FS results show that 3D-printed group exhibited the highest FS (119.11 ± 9.29 MPa), followed by the conventional group (95.28 ± 5.59 MPa) and milled group (93.01 ± 6.27 MPa)^7,35,36,45,52^. However, this advantage was short-lived, as the FS of 3D-printed materials deteriorated over time, ultimately dropping to nearly two-thirds of its initial value after 24 months, resulting in lower strength than both the milled and conventional materials^35^.

Regardless of thermocycling, 3D-printed group exhibited a statistically significant higher FS (*p < 0.05*) than conventional and milled materials^7^. Also, the 3D-printed *Freeprint* denture material showed the highest value of (103.33 MPa), followed by *Meliodent* heat cure denture base material (97.06 MPa), and *Polident Pink* CAD/CAM milled material (96.27 MPa)^45^. It was also reported that the 3D-printed materials *Dentica* Denture Base II and High Impact Denture Base demonstrated higher FS than the milled *Ivotion* denture base material^36^. Additionally, it was provided that the highest flexural strength values were in 3D-printed group (113.53 ± 7.94 MPa), followed by the milled (104.65 ± 5.12 MPa) and the conventional group (232.67 ± 32.60 MPa)^52^.

#### 3D-printed vs. conventional materials

Conventional heat-polymerized materials generally exhibited moderate FS with values higher than the majority of 3D-printed materials but remained inferior to milled materials in most comparisons^26,35,42,43^. However, A few studies reported that 3D-printed materials exhibited higher FS than conventional heat polymerized denture base materials^40,50,52^. In one study, the 3D-printed material achieved a FS of (87.34 ± 6.39 MPa), surpassing the conventional material, which scored (80.79 ± 7.64 MPa)^40^. Once again, the highest mean value (123.8 MPa) was achieved with *V-Print Dentbase* 3D-pinted material, while *Villacryl Rapid* conventional heat polymerized showed the lowest mean value (101.8 MPa) showing actually no statistically significant (*p = 0.527*) differences^50^.

### Surface hardness

Nineteen studies evaluated the surface hardness of 3D-printed denture base materials compared to other manufacturing methods (Table 4).

**Table 4:** Hardness values and study settings for included studies.

Study ID	Groups (method of fabrication + brand of resin material)	Resin brand	3d Printing parameters			Hardness Mean ± SD	
			Layer thickness (µm)	Building oriantaion	post-curing time	Mean	SD
^27^	3D (Nextdent)	Nextdent^®^ base; Vertex-Dental B.V., Vertex Global Holding, AV Soesterberg, Netherlands	50	45°	15 min	42.17	0.66
	M (M-PM)	Pre-polymerized PMMA pucks (M-PM™ disc, Merz Dental GmbH, Lütjenburg, Germany)				45.09	1.24
	H(Acrostone)	Acrostone heat cure denture basematerial, Egy				42.86	0.28
^16^	3D (Formlabs)	Denture Base Resin LP Formlabs Inc., MA, USA	50	45°	30 min	16.351	0.842
	M (Merz)	Prepolymerized blanks M-PM, Merz Dental GmbH, Lütjenburg, Germany				18.035	0.702
^29^	H (Ivoclar)	ProBase^®^ Hot (Ivoclar Vivadent AG)	100			17.39	0.21
	C (Ivoclar)	ProBase^®^ Cold (Ivoclar Vivadent AG)				16.98	0.24
	M (Ivoclar)	Ivoclar Vivadent AG				13.75	0.22
	3D FDM (Material4print) x	Material4print		0°		10.41	0.19
	3D FDM (Material4print) y			90		10.16	0.11
	3D FDM (Material4print) z					9.16	0.19
	3D SLA GR (Formlab) x	Formlabs Inc.		0°		5.65	0.24
	3D SLA GR (Formlab) y			90°		5.49	0.02
	3D SLA GR (Formlab) z					5.05	0.24
	3D SLA DR (Formlab) x			0°		13.2	0.29
	3D SLA DR (Formlab) y			90°		19.21	0.98
	3D SLA DR (Formlab) z					13.67	0.63
^32^	3D (Asiga DentaBASE)	Asiga DentaBASE, Sydney, Australia	100	0°	10 min	13.76	2.22
	M (Ivotion Base)	Ivotion Base, Ivoclar Vivadent, Schaan, Liechtenstein				14.6	0.36
	T (Polyan IC)	Polyan IC, Bredent, Senden, Bayern, Germany				20.05	1.13
	I (SR-Ivocap)	SR-Ivocap, Ivoclar Vivadent, Schaan, Liechtenstein				16.56	0.9
^33^	H (Clássico)	Classic resin	50	45°	10 min	22.33	0.76
	M (Blue Dent)	Blocks of medium-pink BlueDent Ltd.				24.32	1.04
	3D (Smart Dent)	Medium-pink liquid resin SmartDent Ltd.				20.44	1.32
^35^	H (Onda Cryl)	(medium pink color, Onda Cryl, Clássico)				21.64	0.85
	Mi (Onda Cryl)	(medium pink color, Onda Cryl, Clássico)				20.95	0.47
	M (Blue Dent)	(medium pink color, BlueDent, Articon)				21.35	0.41
	3D (Liquid resin)	(medium pink color, SmartDent)				17.45	1.1
^37^	M (G-CAM)	G-CAM; Graphenano DENTAL SL (GC)		0°		28.31	1.62
	M (Ivotion Base)	Ivotion Base; Ivoclar Vivadent AG (IV)				21.45	1.3
	3D (Denturetec)	Denturetec; Saremco (DT)				16.46	2.65
^43^	H (Major.Base.20)	(Major.Base.20)	50	90°		41.63	2.03
	3D (NextDent)	(NextDent)				34.62	2.1
^44^	M	(M-PM; Merz Dental GmbH)	50	45°		18.02	0.67
	3D	(Denture base RP; Formlabs)			30 min	16.26	0.79
^45^	Meliodent heat cure	Denture base material, PMMA, heat cured				20.58	0.52
	Vertex Thermosens	Denture base material, polyamide, injection technique				10.61	0.24
	Ivobase CAD pink V	CAD-CAM denture base material, subtractive manufacturing				17.23	0.99
	Polident pink CAD-CAM disc basic	CAD-CAM denture base material, subtractive manufacturing				22.86	0.72
	Anaxdent pink blank U medium pink	CAD-CAM denture base material, subtractive manufacturing				18.83	0.48
	Freeprint denture	CAD-CAM denture base material, additive manufacturing				21.3	0.45
	Imprimo LC denture	CAD-CAM denture base material, additive manufacturing				16.55	0.81
^2^	H	Major.Base.20, Major Prodotti Dentari, Moncalieri, Italy	50	90°		39.6	9.9
	AvaDent	AvaDent denture base puck (AvaDent, Global Dental Science Europe, Tilburg, The Netherlands)				46.3	2.9
	IvoCad	IvoBase CAD (Ivoclar Vivadent, Schaan, Liechtenstein)				46.9	5.9
	3D (ASIGA)	ASIGA DentaBase, (Asiga pty Ltd, Alexandria, Australia)				31.1	7.5
	3D (FormLabs)	FormLabs Denture Base LP (FormLabs, Somerville, MA, USA)				17.5	2.8
	NextDent	Denture 3D+ (NextDent B.V., Soesterberg, The Netherlands)				15.2	0.15
^55^	H (Major.Base.20)	(Major.Base.20, Major Prodotti Dentari, Moncalieri, Italy. Shade: light pink)	50	90°		46.29	6.22
	Thermoformed Acetal (Bio Dentaplast)	thermoformed acetal (Bio Dentaplast, Bredent, Germany. Shade: white A2)				34.52	4.6
	Thermoformed Polyamide (Flexiultra Sabilex)	Thermoformed polyamide (Flexiultra Sabilex, Buenos Aires, Argentina. Shade: pink 78, orange pink)				21.92	3.26
	M AvaDent	(AvaDent denture base puck, AvaDent, Global Dental Science Europe, Tilburg, The Netherlands. Shade: light pink)				47.99	8.4
	M IvoCad	(Ivoclar Vivadent, Schaan, Liechtenstein. Shade: light pink				30.33	9.61
	3D NextDent	(NextDent B.V., Soesterberg, The Netherlands. Shade: light pink).				20.57	4.51
	3D FormLabs	(FormLabs, Somerville, MA. Shade: light pink				23.2	4.2
^48^	Milled Base	AvaDent Denture base puck (AvaDent, Global Dental Science Europe, Tilburg, The Netherlands)	100			15.94	0.36
	Printed BP1	NextDent Base (Vertex-Dental B.V., Soesterberg, The Netherlands)		0°	10 min	17	1.26
	Printed PB2					6.68	2.26
	Printed PBV2			90°			
^8^	M (Polident)	(Polident d.o.o. VolčjaDraga 42, VolčjaDraga, Slovenia)	100	45°	15 min	26.14	0.39
	3D (NextDent)	(NextDent, Soesterberg, The Netherlands)				20.06	0.39
	Flexible (BreFlex)	(BreFlex Second Edition, Germany)				14.39	0.89
^49^	H PMMA (Acrostone)	Acrostone heat-cured denture base (2023 Acrostone Dental & Medical Supplies. Egypt)	50	90°	10 min	20.055	1.641
	Thermoplastic polyamide (Flexi Ultra)	Flexi Ultra -flexafil s.a.c.i. leopoldo marechal 1312 – Buenos aires—Argentina “orange-pink 78”				12.133	1.173
	M (IvoCad)	IvoCad (Ivoclar Vivadent, Schaan, Liechtenstein)				14.605	1.417
	3D (NextDent)	NextDent Denture 3D+ (NextDent B.V., Soesterberg, The Netherlands)				15.138	1.706
^4^	M (meliodent)	NextDent Denture 3D+(NextDent Denture 3D+; Nextdent B.V., Soesterberg, Nether-lands), Dentona Optiprint Denture 3D Printer resin (DentonaOptiprint Denture 3D Printer resin; Dentona AG., Dort-mund, Germany) and ASIGA DentaBase (ASIGA DentaBase; ASIGA, Sydney, Australia)				18.11	0.65
	3D (Dentona)		50	45°		16.41	0.96
	3D (Asiga)					16.24	0.79
	3D (Next Dent )					16.2	0.93
^50^	3D printed (V-Print Dentbase)	V-Print Dentbase	50			11.6	0.34
	3D printed (Denture 3D+)	Denture 3D+			30 min	12.5	0.29
	H (Probase Hot)	Probase Hot				14.1	0.69
	H (Probase Hot)	Villacryl Rapid				14.9	0.41
^56^	3D (Asiga DentaBASE pink shade (PP))	Asiga	100	0°	10 min	13.76	2.22
	M (Ivotion Base pink shade (MP))	Ivotion Base				14.6	0.36
^3^	conventional heat polymarized	ProBase Ho				131.37	2.94
	conventional heat polymarized	Paladon 65				118.63	3.92
	conventional heat polymarized	Interacryl Hot				129.41	3.92
	cad cam	IvoBase CAD				94.12	10.78
	cad cam	Interdent CC disc PMMA				72.14	2.94
	cad cam	Polident CAD/CAM disc basic				141.18	3.92
	3d printing	NextDent Base				114.71	7.84
	Injection pressing	Vertex ThersmoSens				64.71	10.78

#### 3D-printed vs. milled materials

In almost all studies comparing the hardness of 3D-printed and milled materials, milled materials demonstrated higher hardness^3,8,12,16,34,35,45,57^. Milled materials showed consistently superior surface hardness. For example, results revealed that the Vickers hardness of milled denture base material was higher than that of 3D-printed resins both before (18.02 ± 0.67 vs. 16.26 ± 0.79) Vicker hardness number (VHN) and after thermal cycling (16.26 ± 0.79 vs. 12.42 ± 1.30) VHN^16^.

Milled groups frequently recorded values exceeding 30 VHN, while 3D-printed materials such as *NextDent* ranged from (15.2 ± 0.15) VHN to (34.62 ± 2.1) VHN^8,18,49^depending on the printer and resin formulation. It recorded much lower values (15.2 ± 0.15) VHN and (20.57 ± 4.51) VHN, nearly half the milled and heat-polymerized groups^2^. It also scored (15.138 ± 1.706), slightly above the milled group but below the conventional^49^. *Material4Print* exhibited the lowest surface hardness among 3D-printed materials (10.16 ± 0.11) VHN, significantly lower than both milled and conventional resins^29^.

#### 3D-printed vs. conventional materials

When comparing conventional heat-polymerized materials to 3D-printed ones, the conventional materials generally outperformed 3D-printed materials in surface hardness^6,18,50^. Studies reported values for conventional heat-polymerized resins in the range of (20–28) VHN, whereas most 3D-printed resins demonstrated lower or comparable values, with significant variability depending on resin brand and orientation. For instance, microhardness mean values ranged from 11.6 Knoop hardness number (KHN) for *V-Print Dentbase* to 14.9 KHN for *Villacryl Rapid*, with a statistically significant difference (*p < 0.001*) in microhardness among the tested resins.

Both conventional heat-cured resins exhibited significantly higher microhardness (*p < 0.001*) than the two 3D-printed light-cured resins. Within the same resin type, *Denture 3D +* showed significantly higher microhardness than *V-Print Dentbase* (*p* = 0.003), and *Villacryl Rapid* had significantly higher values than *Probase Hot* (*p* = 0.01)^50^. The highest mean Vickers hardness number (VHN) was recorded for conventional heat-polymerized resin (18.11 ± 0.65), followed by *Dentona* (16.41 ± 0.96) (VHN), *ASIGA* (16.24 ± 0.79) (VHN), and *NextDent* (16.20 ± 0.93) (VHN). The results showed a statistically significant difference (*p ≤ 0.05*) in surface microhardness between the conventional heat-polymerized group and the other tested groups. However, the differences in surface microhardness among the 3D-printed resin groups were statistically nonsignificant (*p > 0.05*)^18^.

### Fracture toughness

Five studies evaluated the fracture toughness of 3D-printed denture base materials compared to other manufacturing methods. The results generally favorited the milled groups over the 3D-printed ones when it came to their fracture toughness values (Table 5). The fracture toughness values, by megapascal times square root of meter (MPa·m¹/²), of the CAD-CAM milled group were superior to those of the 3D-printed group both before (4.16 ± 0.06 MPa·m¹/² and 1.30 ± 0.06 MPa·m¹/², respectively) and after thermocycling (3.82 ± 0.08 MPa·m¹/² and 0.78 ± 0.05 MPa·m¹/², respectively)^16^. The fracture toughness of Formlabs and Dentca (3D‑printed) was notably lower than that of Leucitone 199 (conventional) (*P < 0.05*). In comparison to Avadent (CAD/CAM milled), Leucitone 199 (conventional) demonstrated considerably reduced fracture toughness (*P < 0.05*)^28^.

**Table 5:** Studies on fracture toughness and their results.

Study	Groups (method of fabrication + brand of resin material)	Resin brand	3d Printing information			Fracture Toughness	
			printer machine	Layer thickness (µm)	Building orientation	Mean	SD
^16^	3D (Formlabs)	Denture Base Resin LP Formlabs Inc., MA, USA	Form 3; Formlabs	50	45°	1.276	0.069
	M (Merz)	Prepolymerized blanks M-PM, Merz Dental GmbH, Lütjenburg, Germany				4.138	0.092
^28^	M (RUTHENIUM)	PMMA disc, RUTHENIUM	NEXTDENT 5100			97.679	1.877
	3D (NEXTDENT)	NEXTDENT denture 3D + pink resin.				49.257	0.254
	H (DPI)	Heatcured acrylic DPI				68.631	1.143
^36^	M (Ivotion (Pink))	Ivotion Base Milled Denture Base disc (Ivoclar Vivadent, Schaan, Liechtenstein)	Denture Base II (Dentca, Torrance CA) and High Impact Denture Base (SprintRay, Los Angeles, CA) bars were fabricated using a DLP printer (Pro 55 S, SprintRay). Lucitone Digital Print (Dentsply Sirona, Charlotte, NC) bars were fabricated using a CLIP printer (M3 printer, Carbon, Redwood City, CA). TrueDent Resin (Stratasys, Rehovot, Israel) bars were fabricated using a material jetting printer (J5 DentaJet printer, Stratasys).	100	0°	1.87	0.09
	H (Lucitone 199 (Light))	Lucitone 199 Denture Base Resin (Dentsply Sirona)				2.03	0.12
	3D DLP (Dentca Denture Base II)	Denture Base II (Dentca, Torrance CA)				0.54	0.05
	3D DLP (High Impact Denture Base)	High Impact Denture Base (SprintRay, Los Angeles, CA)				1.76	0.15
	3D CLIP (Lucitone 3D)	Lucitone Digital Print (Dentsply Sirona, Charlotte, NC)				2.01	0.09
	3D Material jetting (TrueDent Resin)	TrueDent Resin (Stratasys, Rehovot, Israel)				0.58	0.05
^39^	3D (from Formlabs)	OP (Original Pink) denture base material (Ref. PKG-RS-F2-DB) from Formlabs (Formlabs, Somerville, MA, USA).	(Formlabs, Somerville, MA, USA)		0°	6.304	2.6
	M (from Formlabs)	(Original Pink) denture base material (Ref. PKG-RS-F2-DB) from Formlabs (Formlabs, Somerville, MA, USA)				8.634	1.225
^44^	M	(M-PM; Merz Dental GmbH)	(Form 3; Formlabs)	50	45	4.16	0.06
	3D	(Denture base RP; Formlabs)				1.3	0.06
^48^	M (AvaDent)	AvaDent Denture base puck (AvaDent, Global Dental Science Europe, Tilburg, The Netherlands)	rotary table saw (Inca, Injecta, Teufenthal, Switzerland) equipped with a 3 mm thick stainless circular blade (Oertli Werkzeuge, H ¨ori, Switzerland)	100		794.322	65.17
	3D BP1 (NextDent Base)	NextDent Base (Vertex-Dental B.V., Soesterberg, The Netherlands)	Rapid Shape D30, Rapid Shape GmbH, Heimsheim, Germany)		0°	408.038	262.94
	3D PB2 (NextDent Base)		a third-party 3D-printer Form 2, Formlabs, Massachusetts, US			271.334	192.55
	3D PBV2 (NextDent Base)		Rapid Shape D30, Rapid Shape GmbH, Heimsheim, Germany)		90°	414.05	161.85

### Impact strength

Only four studies evaluated the impact strength of 3D-printed denture base materials compared to other manufacturing methods (Table 6). The values of impact strength for 3D-printed groups, measured by (kilojoule per square meter) kJ/m², generally ranged somewhere between 6.32 ± 0.50 and 2.44 ± 0.31 KJ/m^2^ before and after thermocycling treatment^43^. The results revealed the milled group exhibited a greater mean impact strength than the 3D-printed group both without (*P = 0.004*) and with thermocycling (*P = 0.050*). Nonetheless, thermocycling did not significantly influence the impact strength of either group (*P > 0.05*)^8,41^.

**Table 6:** Studies on impact strength and their results.

Study	Groups (method of fabrication + brand of resin material)	Resin brand	3d Printing information			Impact Strength	
			printer machine	Layer thickness (µm)	Building orientation	Mean	SD
^29^	H (Ivoclar)	ProBase^®^ Hot (Ivoclar Vivadent AG)	(LulzBot TAZ 6; Aleph Objects Inc.), using a 3D filament of PMMA (Material4print), (Form 2; For-mlabs Inc) for SLA group	100		1.39	0.06
	C (Ivoclar)	ProBase^®^ Cold (Ivoclar Vivadent AG)				1.47	0.1
	M (Ivoclar)	Ivoclar Vivadent AG				1.77	0.02
	3D FDM (Material4print) x	Material4print			0°	2.07	0.23
	3D FDM (Material4print) y				90°	2.36	0.18
	3D FDM (Material4print) z				45	0.93	0.04
	3D SLA GR (Formlab) x	Formlabs Inc.			0°	1.65	0.05
	3D SLA GR (Formlab) y				90°	1.59	0.04
	3D SLA GR (Formlab) z				45	1.68	0.05
	3D SLA DR (Formlab) x				0°	2.45	0.08
	3D SLA DR (Formlab) y				90°	2.27	0.05
	3D SLA DR (Formlab) z				45	2.14	0.03
^41^	M (Merz Dental GmbH)	(M-PM; Merz Dental GmbH)	(Form 2; For mlabs)		45	2.33	0.73
	3D (Formlabs)	Denture Base RP; Formlabs)				0.39	0.18
^43^	H (Major.Base.20)	(Major.Base.20)	(NextDent; 3D systems, Vertex Dental B.V., Soester berg, Netherland)	50	90°	6.32	0.5
	3D (NextDent)	(NextDent)				2.44	0.31
^8^	M (Polident)	(Polident d.o.o. VolčjaDraga 42, VolčjaDraga, Slovenia)	3D printer (Phrozen Shuffle, Phrozen, Hsincu City, Taiwan)	100	45°	25.88	0.54
	3D (NextDent)	(NextDent, Soesterberg, The Netherlands)				23.18	0.71
	p (BreFlex)	(BreFlex Second Edition, Germany)				30.3	0.83
^4^	M (meliodent)		(Asiga MAXTM; ASIGA, Sydney, Australia)	50	45°	16.76	1.75
	3D Dentona	Dentona Optiprint Denture 3D Printer resin (DentonaOptiprint Denture 3D Printer resin; Dentona AG., Dort-mund, Germany)				17.98	1.76
	3D Asiga	ASIGA DentaBase (ASIGA DentaBase; ASIGA, Sydney, Australia)				16.76	1.75
	3D Next Dent	NextDent Denture 3D+(NextDent Denture 3D+; Nextdent B.V., Soesterberg, Nether-lands)				15.2	0.69

## Discussion

The evolution of denture base fabrication has significantly progressed with the introduction of 3D printing technologies. This systematic review compared the mechanical properties of 3D-printed denture base materials with conventional heat-polymerized and CAD-CAM milled alternatives. The findings revealed substantial differences in FS, surface hardness, fracture toughness, and impact strength, which are critical determinants of clinical performance. Variations in FS and surface hardness directly impact clinical longevity, resistance to fracture, and patient satisfaction regarding many dental materials^58^.

With a primary focus on material composition, fabrication procedures, post-processing methods, and testing protocols, this review examined a number of probable sources of heterogeneity among the study outcomes. The composition of the material became an important consideration, as different resin compositions resulted in vast differences in mechanical performance^59^. For example, compared to Material4Print (10.16 VHN), 3D-printed resins like NextDent and ASIGA showed higher hardness values (15–34 VHN)^58^. Also, 3D-printed resins displayed variability based on photoinitiator systems and monomer conversion rates, whereas milled materials showed more consistency because of their standardized pre-polymerized PMMA blocks^60^.

Heterogeneity was also influenced by the parameters and methods of fabrication^61^. The orientation of printing was important; specimens printed horizontally had greater FS because of the best layer alignment, while specimens printed vertically had anisotropic weaknesses^62^. Results were also affected by layer thickness; bigger layers (100–200 μm) increased durability but ran the risk of interlayer inconsistencies, whereas thinner layers (25–50 μm) increased resolution but decreased impact strength^63^.

Another important factor was post-curing procedures; prolonged UV curing (60 min, for example) improved FS and hardness by strengthening polymer cross-linking, however excessive curing occasionally made some resins more brittle^64^. Variability was further affected by environmental conditions and post-processing. Compared to milled or conventional PMMA, thermocycling considerably accelerated the degradation of 3D-printed materials, resulting in FS decreases of up to 33% over time^16,65^.

For milled materials, which benefited from high-pressure polymerization, showed no leftover monomers, residual monomers in 3D-printed resins functioned as plasticizers, reducing mechanical characteristics^11,12^. Discrepancies were also brought about by testing procedures, as disparities in equipment and standards (such as ISO 1567 for FS) among research resulted in findings that were not consistent. For instance, because specimen geometry and load applications varied, impact strength values varied from 2.44 to 6.32 kJ/m²^66^.

Another degree of complication was introduced by geographic and manufacturer diversity; research conducted in places like Saudi Arabia and Egypt frequently used locally accessible resins, but studies conducted in other places (such the USA and Brazil) tested commercial brands like AvaDent or Freeprint, with varying results.

The sensitivity analysis, which concluded that milled materials consistently showed improved mechanical properties, validated the findings’ robustness. Material and methodological differences were the main causes of the variation between investigations, highlighting the necessity of standardized procedures in further studies to reduce variability and improve comparability. These insights are critical for clinicians and researchers aiming to optimize denture base fabrication techniques and material selection for long-term clinical success.

### Flexural strength

A high flexural strength (FS) of resin base material is critical for denture durability and for preventing failure under load^67,68^. Throughout its lifespan, a denture is subjected to repetitive masticatory forces, which can eventually result in cracking, fractures, or complete failure^69^. Such failures are exacerbated by factors like poor fit, improper design, or the presence of notches^70^. FS indicates how resistant a material is to fracture and offers some predictability regarding its behavior under static loads; thus, elevated values of this mechanical property are clinically significant for decreasing the incidence of fractures in a prosthetic base^71^.

FS testing measures the maximum stress a material can endure before yielding and reflects the combined tensile, shear, and compressive strengths of the material^72^. The three-point flexure test, endorsed by ISO, is the standard method for assessing the FS of polymers^73^, with a clinically acceptable threshold set at no less than 65 MPa (ISO 1567) for a denture base material^73^. The majority of studies assumed that milled restorations exhibit higher FS compared to 3D-printed ones, with each study providing its own explanation for the results^2,26,31,34,40,41^. The chemical makeup of the photo-polymerized resin employed in 3D-printing and the structure of the final product may be a contributing factor to the reduced FS outcomes.

The decreased FS of 3D-printed resins in contrast to milled ones could stem from its layer-based manufacturing, post-curing conditions, and the presence of leftover monomer^2^. The leftover monomer functions as a plasticizer, which diminishes the FS of the produced denture base^26^. On the other hand, The increased strength of milled materials may have been linked to the ideal pressure and temperature conditions during the polymerization of the milled resin, resulting in a highly cross-linked structure that are dense, with minimal shrinkage, porosity, or free monomers^41^.

Generally, both milled and 3D-printed resins experienced a notable reduction in FS post-thermocycling, but the 3D-printed samples deteriorated at a faster rate than the milled ones^74^. The deterioration of the 3D-printed samples may have been connected to residual stress from water uptake and temperature fluctuations, which can cause the layers in a 3D-printed item to separate, leading to fractures and long-term structural issues^75^. Multiple studies provided rationale for these outcomes via the internal composition of the 3D-printed materials: the resin from the research group possesses a reduced conversion of monomer to polymer, potentially influencing the mechanical properties of the substance^56^.

To begin, *Gad et al.*^43^ interestingly explored regarding the connection between consecutive layers of the 3D-printed resins, noting that stratification in a direction aligned with the load’s direction may lead to inadequate adhesion and, as a result, exert a detrimental effect on the layer’s own resistance. Researchers discovered empty spaces at the fractured locations of the printed specimens as well, therefore these areas were recognized as elements that lead to the reduction in mechanical efficiency. The presence of voids in printed resins may be attributed to the possible inclusion of air voids/bubbles during the layered printing of the specimen as well, which influences the strength of the specimens^43^. These gaps can weaken the interfacial bonding layers, resulting in delamination and the onset of fractures.

Only a few studies reported higher results for their tested 3D-printed materials compared to either conventional or milled bases. The 3D-printed Temp Print composite tested demonstrated encouraging outcomes, exhibiting the highest FS among the pink resin^38^. Another study reported superior FS values of the 3D-printed resin bases amongst all the groups^52^. They explained that the post-curing duration they applied was 30 + 30 min, which exceeds the post-curing time reported in comparable studies within the literature clarifies why the 3D-printed samples showed greater FS^52^. This is based on the fact that as the total curing duration extends, the conversion of double bonds rises as well.

Therefore, lengthening the final curing duration can enhance the mechanical characteristics of 3D-printed denture-base materials, and eventually the FS of materials made through 3D printing rises with extended curing time^76^. SLA and DLP are the main technologies for denture base fabrication. One study explored FDM^29^ (Table 2).

The mechanical characteristics of 3D-printed resins are influenced by build parameters, orientation angle during construction, the post-curing procedure^77,78^ the software used, the number and thickness of layers, and shrinkage occurring between layers^79–81^. The printing settings are typically established by the manufacturer and can only be adjusted for layer thickness (layer height) and printing orientation. The permissible layer thickness is 100 μm and varies between 25 and 200 μm^79^. Nonetheless, the strength of the 3D-printed item improves as the layer thickness diminishes, thanks to enhanced resin curing and minimized dimensional alterations^81,82^. Therefore, careful optimization of these parameters is critical for producing clinically durable prostheses.

Printing orientation is a critical manufacturing parameter that significantly influences the mechanical properties of 3D-printed objects^83,84^. Recent systematic reviews have reported that FS is notably higher when printing is performed at a horizontal orientation^85^.The superior FS observed in specimens printed horizontally can be attributed to the alignment of layers perpendicular to the applied forces, enhancing mechanical integrity. Conversely, tilted and upright printing orientations have been shown to be less effective in improving mechanical properties^85^. However, in the included studies, the majority of printing angles were 90 (Table 2).

Furthermore, longer post-curing durations have demonstrated improvements in the FS of specimens printed at standard orientations (0°, 45°, and 90°), particularly when employing stereolithography (SLA) and digital light processing (DLP) technologies. This underscores the intricate relationship between post-processing protocols and printing orientation in optimizing the mechanical performance of denture base materials. These findings are consistent with our subgroup analysis, which demonstrated higher FS values in studies using 0° printing orientation. This supports the interpretation that build direction significantly influences performance outcomes and may contribute to the observed heterogeneity in results.

### Surface hardness

Another essential mechanical property influencing wear resistance, microbial adhesion, and overall longevity of denture bases is surface hardness, which shows the degree of a material’s resistance to surface plastic deformation. Consequently, the hardness of acrylic indicates the likelihood of deterioration of the polymer matrix. As a result, with a decrease in hardness, the matrix deteriorates, raising the likelihood of material fracture, along with the possibility of microbial retention and pigmentation. As a result, the lifespan of the denture base reduces^86^.

Harder surfaces resist mechanical wear, reduce biofilm accumulation, and maintain color stability over time^87^. Milled denture bases exhibited the highest surface hardness, with values consistently exceeding 30 VHN, making them the most resistant to wear and surface degradation. Milled PMMA achieves superior hardness due to high-density polymerization and cross-linking in pre-polymerized pucks, minimizing porosity and residual monomer content^2,16,29^. Conventional heat-polymerized PMMA showed moderate hardness, typically in the range of 20–28 VHN, surpassing most 3D-printed resins.

Conventional heat-polymerized resins undergo adequate polymerization under heat and pressure, but their hardness is lower than milled materials due to polymerization shrinkage and potential porosities^6,18,50^. 3D-printed denture bases displayed a wide range of hardness values (10–34 VHN), depending on resin formulation, layer thickness, and post-curing conditions^87^. 3D-printed materials, especially those using SLA/DLP, tend to have lower hardness due to incomplete polymerization, layering defects, and residual monomer presence, resulting in increased susceptibility to surface degradation and bacterial colonization^88^.

Many printing parameters have an effect on hardness. Post-curing duration and UV exposure significantly influence the hardness values. The post-curing time is directly correlated with the surface hardness of the material, which is attributed to the degree of conversion. Prolonged post-curing enhances polymer cross-linking, improving hardness^89^. Post-curing plays a more dominant role than printing orientation^90,91^. Furthermore, resin composition plays a major role, with some photopolymerized resins approaching conventional hardness values, while others remain inferior.

All printing parameters must be optimized to obtain the best orientation for utilizing printed resin parts with optimum properties. Applying 10 min. post-curing showed higher hardness at 0°^92^whereas 45° and 90° orientations showed improved hardness when low-viscosity resins were used. Low-viscosity resins showed higher overall hardness than high-viscosity resins, irrespective of the layer thickness and build-up angle^93^. Moreover, thinner layers (< 50 μm) improve resolution but may reduce hardness due to increased light scattering and potential oxygen inhibition at the layer surface. Thicker layers (> 100 μm) enhance durability but can introduce layering inconsistencies, affecting overall hardness^6^.

### Fracture toughness

Fracture toughness is a critical property determining a material’s ability to resist crack propagation under stress^94^. Higher fracture toughness reduces the risk of sudden denture fractures, particularly in patients with high occlusal forces. Milled denture bases exhibited the highest fracture toughness, with values exceeding 4.16 ± 0.06 MPa m¹/², making them the most resistant to crack propagation. Milled PMMA has superior fracture toughness due to its high-density polymer structure and elimination of internal defects owing to the pre-polymerization of the PMMA blocks resulting in better intermolecular bonding and fewer voids, leading to superior toughness^74^.

Conventional heat-polymerized PMMA demonstrated moderate fracture toughness, superior to 3D-printed materials but still lower than milled resins, benefiting from high-pressure polymerization. However, these conventional resins are still prone to processing defects such as porosities and residual stresses^95^. 3D-printed denture bases on the other side, had significantly lower fracture toughness, with values ranging from 0.78 to 3.82 MPa m¹/², indicating higher susceptibility to chipping and crack formation. Formlabs and Dentca (3D-printed) had notably lower toughness compared to heat-polymerized and milled resins. Leucitone 199 (conventional) outperformed some CAD-CAM milled resins, demonstrating that polymerization technique influences toughness^96^.

Regarding 3D-printed resins, printing parameters are among the most influential factors affecting the resin fracture toughness, primarily due to layering defects caused by additive manufacturing, which weaken interlayer bonding. Incomplete polymerization in photopolymerized resins (SLA, DLP leads to weaker molecular chains. Presence of voids and micro-cracks could also reduce the resistance to stress and impact forces. Thicker layers (> 100 μm) may increase fracture resistance by reducing weak interlayer zones. Thinner layers (< 50 μm) improve detail accuracy but may introduce brittleness due to increased polymerization inconsistencies.

Horizontally printed specimens generally exhibit higher fracture toughness, as interlayer bonds align better with applied forces. Vertically printed specimens are more prone to crack propagation along layer interfaces. Extended UV and heat curing improve polymer cross-linking, thus enhancing fracture toughness. Insufficient post-curing leads to weak polymer chains, reducing fracture resistance^97^.

### Impact strength

Impact strength is especially important in elderly or disabled populations where accidental dropping of prostheses is common or those with high bite forces. A denture with low impact strength is more likely to fracture upon sudden stress, leading to frequent repairs or remakes. Milled denture bases demonstrated the highest impact strength due to their dense polymer network and absence of layering defects, which allows them to absorb and distribute forces effectively, making them the most resistant to sudden forces and accidental drops^98^.

Conventional heat-polymerized PMMA exhibited moderate impact strength, superior to most 3D-printed materials but still lower than milled resins. 3D-printed denture bases had the lowest impact strength, with values ranging from 2.44 to 6.32 kJ/m², indicating higher susceptibility to sudden fractures. Conventional PMMA exhibits moderate impact strength, benefiting from cross-linked polymerization but still prone to stress concentrations from polymerization shrinkage^66^.

The lower values for 3D-printed bases (as low as 2.44 kJ/m²) suggest limited resilience under sudden impact. Build orientation and layer thickness significantly affect this property. Horizontally printed specimens exhibit higher impact strength due to better interlayer bonding and even stress distribution. Vertically printed specimens are more prone to fractures along layer interfaces. Thicker layers (> 100 μm) may slightly improve impact resistance by reducing the number of weak interlayer zones. Thinner layers (< 50 μm) increase resolution but may reduce impact resistance due to a higher number of layers with weak bonding. Extended UV and heat curing enhance polymer cross-linking, improving impact resistance. Inadequate post-curing leaves residual monomers, leading to reduced toughness and increased brittleness^6^.

Milled PMMA remains the best option for long-term dentures, particularly for patients with a high risk of dropping their prosthesis. 3D-printed denture bases require modifications to improve impact resistance, such as reinforcement with nanoparticles or hybrid polymer formulations. Conventional PMMA is a suitable alternative, but it may require reinforcement with impact modifiers to improve performance^8,99^. These findings underscore the need for material and protocol optimization in 3D printing for long-term prosthesis success.

Milled PMMA exhibited the highest impact resistance, followed by conventional heat-polymerized resins, while 3D-printed resins were the most brittle. Thermocycling had a significant effect on the impact strength of 3D-printed resins, further reducing their performance over time. Some high-performance 3D-printed materials showed impact strength values approaching those of conventional PMMA, but overall, most 3D-printed materials remain more fragile due to layer-by-layer fabrication, which introduces weak interlayer bonding that compromises energy absorption. Incomplete polymerization may also lead to a lower cross-linking density and increased brittleness. Furthermore, the presence of micro-cracks and voids could act as fracture initiation points under sudden impact^6^.

### Clinical relevance

The mechanical properties of denture base materials, particularly flexural strength (FS) and surface hardness are directly related to clinical performance, longevity, and patient satisfaction. FS is crucial for withstanding masticatory forces and preventing midline fractures, which are a common mode of failure in complete dentures. Studies have reported that up to 63% of denture fractures occur within the first three years of use, often due to inadequate mechanical strength. Materials with higher FS, such as milled PMMA, are less likely to fracture under functional loads or accidental drops, contributing to longer service life and fewer repairs. In contrast, 3D-printed bases, with their wider variability and generally lower FS, may be more susceptible to such failures, especially in patients with high bite forces or parafunctional habits.

Surface hardness is also clinically significant, as it affects wear resistance, hygiene, and esthetics. Harder denture bases resist scratching and abrasion from cleaning, maintaining smooth surfaces that discourage microbial adhesion and biofilm formation. This is important for patient comfort, oral health, and the prevention of staining or odor. Softer materials may become rougher over time, increasing the risk of microbial colonization and compromising hygiene. The results, which show milled bases consistently outperforming 3D-printed ones in hardness, suggest that milled materials are preferable for long-term use, while 3D-printed bases may be suitable for interim prostheses or cases where rapid fabrication is prioritized.

### Clinical implications

- Milled denture bases remain the gold standard for patients requiring durable, long-term prostheses due to their high strength, wear resistance, and fracture toughness.
- 3D-printed dentures may be suitable for temporary or interim prostheses but require material modifications to improve durability.
- Surface hardness directly impacts microbial adhesion, rougher 3D-printed materials may promote biofilm formation, necessitating optimized post-processing protocols.
- Based on current evidence, 3D-printed denture bases may be suitable for interim use or situations requiring rapid fabrication. However, their long-term clinical performance remains inferior to milled bases, except for selected high-performing materials in specific scenarios.

## Challenges in 3D-printed denture base fabrication

Despite technological advancements, the integration of 3D printing for denture base fabrication faces several challenges that hinder its clinical adoption as a long-term solution. These challenges can be categorized into material limitations, mechanical performance, processing inconsistencies, and clinical viability.

### Mechanical limitations

**Lower flexural strength & fracture toughness**:

- Most 3D-printed denture bases fail to match the strength of milled or conventional PMMA, leading to a higher risk of midline fractures under cyclic loading.
- The layer-by-layer fabrication process introduces weak interlayer bonding, reducing overall durability.

**Brittleness & reduced impact strength**:

- 3D-printed denture bases often lack the toughness needed to withstand accidental drops, making them more susceptible to fractures upon impact.
- Thermocycling studies show that impact strength deteriorates faster in 3D-printed resins compared to milled PMMA.

**Inconsistent surface hardness & wear resistance**:

- Variations in post-curing time and layer thickness affect hardness, impacting the wear resistance and microbial adhesion of the denture.
- Softer surfaces tend to promote plaque accumulation, increasing the risk of biofilm formation and staining.

### Printing & post-processing challenges

**Incomplete polymerization & residual monomers**:

- Photopolymerized 3D-printed resins exhibit a lower degree of polymer conversion, leading to the presence of residual monomers that can compromise biocompatibility.
- Extended post-curing is required to improve hardness, but excessive curing can increase brittleness.

**Effects of printing orientation & layer thickness**:

- Printing at a 90° angle generally yields higher flexural strength, but certain orientations lead to anisotropic properties, making the denture weaker in specific stress directions.
- Layer thickness inconsistencies create microvoids, which reduce fracture resistance and contribute to early material failure.

**Resin composition variability**:

- Different manufacturers use proprietary resin formulations, leading to variability in mechanical properties and compatibility with post-processing protocols.
- Standardization of resin formulations is still lacking in the industry.

### Biocompatibility & clinical limitations

**Cytotoxicity concerns due to residual monomers**:

- Some 3D-printed resins release unpolymerized monomers, which can cause mucosal irritation, allergic reactions, and cytotoxicity.
- Biocompatibility studies are needed to assess the long-term effects of these materials in the oral environment.

**Dimensional stability & fit issues**:

- Milled denture bases provide superior accuracy and fit, while 3D-printed dentures may shrink or distort during post-processing.
- Thermal cycling studies indicate that printed dentures experience higher volumetric changes, affecting occlusion and adaptation over time.

**Microbial adhesion & hygiene maintenance**:

- The higher porosity of 3D-printed denture bases increases microbial adhesion, which can lead to denture stomatitis and infections.
- Chemical denture cleansers may further degrade the surface properties, reducing hardness and structural integrity over time.

### Regulatory & standardization issues

**Lack of standardized testing protocols**:

- Studies use different testing methods and parameters, leading to inconsistent findings regarding mechanical performance.
- ISO 20795-1 standards for denture base polymers were originally designed for heat-polymerized PMMA, and updated guidelines for 3D-printed resins are needed.

**Limited long-term clinical studies**:

- Most research on 3D-printed denture bases remains limited to in vitro studies, with few long-term clinical evaluations available.
- Patient-centered studies evaluating wear resistance, occlusal performance, and fracture rates over time are essential.

## Future directions to overcome challenges

To improve the clinical viability of 3D-printed denture bases, future research should focus on:

1. Developing high-performance resin formulations incorporating nanoparticles (e.g., zirconia, TiO₂, graphene) to enhance mechanical strength and toughness.
2. Optimizing post-curing protocols to maximize polymer conversion while maintaining adequate fracture resistance.
3. Advancing hybrid fabrication techniques, such as combining 3D printing with post-milling or heat polymerization, to improve mechanical stability.
4. Conducting long-term clinical trials to evaluate wear resistance, microbial adhesion, and patient-reported outcomes.
5. Future research should focus on developing standardized mechanical testing protocols, exploring new resin chemistries, and optimizing 3D printing parameters. Interdisciplinary collaboration among materials scientists, engineers, and prosthodontists will be crucial for advancing the field.

## Limitations

- High heterogeneity among included studies.
- Variability in resin formulations and testing standards.
- In vitro nature of most studies.
- Potential publication bias.
- Sample size constraints.

While the meta-analysis offers meaningful comparisons, generalizability to clinical practice is limited due to the in vitro nature of the studies included. Furthermore, the rapid increase in publications on 3D-printed prosthetics may introduce publication bias, favoring studies with positive outcomes. Standardized mechanical testing protocols and clinical trials are needed to validate in vitro findings.

This review’s strengths include a comprehensive search strategy, rigorous risk of bias assessment, and robust meta-analytic methodology. The findings provide clinically relevant insights for optimizing denture base fabrication.

## Conclusion

This systematic review confirms that milled denture bases outperform 3D-printed and conventional PMMA in mechanical performance, particularly in flexural strength, surface hardness, and fracture toughness. While 3D printing offers advantages in efficiency, customization, and material conservation, its mechanical limitations hinder its widespread adoption for permanent prostheses. Advances in resin formulations, post-curing methods, and reinforcement strategies are essential to improve its clinical viability. Key conclusions of this review are: -.

- Milled PMMA remains the superior choice for long-term denture durability due to its high mechanical strength and wear resistance.
- 3D-printed denture bases require material optimization and enhanced post-processing techniques to compete with milled alternatives.
- Future research should focus on integrating nanotechnology and hybrid processing techniques to enhance the strength and longevity of 3D-printed dentures.

## Supplementary Information

Below is the link to the electronic supplementary material.

Supplementary Material 1

## Data Availability

All data generated or analyzed during this study are included in this published article. Additional datasets used and/or analyzed during the current study are available from the corresponding author on reasonable request.
