# Supplementary material for: Systematic review and meta analysis of mechanical properties of 3D printed denture bases compared to milled and conventional materials
Source: Sci Rep. 2025 Aug 9;15:29207. doi: 10.1038/s41598-025-14288-2 (PMC12335478; doi:10.1038/s41598-025-14288-2)
Supplement: Supplementary file 1 — Supplementary Material 1 [file 41598_2025_14288_MOESM1_ESM.docx]

# Supplementary Materials:

# Table S1: Complete Search Strategies Used in Each Database

| Database | Search String / Query | Filters Applied |
| --- | --- | --- |
| PubMed | ("3D printing" OR "additive manufacturing" OR "digital fabrication") AND ("denture base" OR "denture resin") AND ("mechanical properties" OR "flexural strength" OR "hardness") | English, Full-text, 2010-2025 |
| Scopus | TITLE-ABS-KEY(("3D printing" OR "additive manufacturing") AND ("denture base" OR "denture resin") AND ("mechanical properties" OR "flexural strength" OR "hardness")) | English, Articles, 2010-2025 |
| Web of Science | TS=("3D printing" OR "additive manufacturing") AND TS=("denture base" OR "denture resin") AND TS=("mechanical properties" OR "flexural strength" OR "hardness") | English, Articles, 2010-2025 |
| Embase | ('3d printing' OR 'additive manufacturing') AND ('denture base' OR 'denture resin') AND ('mechanical properties' OR 'flexural strength' OR 'hardness') | English, Human, 2010-2025 |

**Data extraction Process**

Data extraction was conducted independently by two reviewers. Disagreements were resolved through discussion or consultation with a third reviewer. No automation tools were used in the data extraction process.

**Excluded studies with justification.**

Several studies were excluded from this review as they did not meet the predefined inclusion criteria focused on the mechanical properties and biocompatibility of denture base materials. For instance, *Choi* et al.^1^, *Çakmak* et al.^2^, and *Takhtdar* et al.^3^ were excluded because their investigations centered on color stability and surface roughness following exposure to staining agents or denture cleansers, outcomes which fall outside the mechanical scope of this review. Although *Alzaid* et al.^4^ reported flexural strength and hardness data, the study was excluded because it primarily examined the influence of salivary pH on material properties rather than baseline comparisons between denture base materials. Furthermore, *Grachev* et al.^5^ was excluded despite reporting mechanical properties, as the testing involved full-scale structural performance of complete dentures, including artificial teeth, rather than isolated evaluation of denture base materials according to standardized mechanical testing protocols. Similarly, *Janyaprasert* et al.^6^ was excluded because the study assessed tensile bond strength between denture base materials and soft liners, focusing on adhesive properties rather than intrinsic mechanical or biocompatibility characteristics of the denture base materials themselves. Lastly, the study by *Jiang* et al.^7^ was excluded as it concentrated on the additive manufacturing process and dimensional accuracy of printed complete dentures, without evaluating mechanical properties or biocompatibility outcomes pertinent to this review. Ravalec et al.^8^ was incomplete data. *Mann* et al.^9^ was to be included as it assessed fracture toughness, but it didn’t measure a baseline measurement. *Tzeng* et al. ^10^ The study evaluates only newly formulated urethane acrylate-based 3D-printed resins and does not include a comparison with conventional heat-polymerized or widely used commercial denture base materials as required by the review’s inclusion criteria.

**Supplementary Table: GRADE Summary of Findings**

**Comparison**: 3D-printed denture base resins vs. CAD-CAM milled and conventional heat-polymerized resins

**Population**: In vitro studies on denture base materials

**Table S2:** GRADE Summary of Findings for Mechanical Properties of Denture Base Materials

| **Outcome** | **No. of Studies** | **Participants (specimens)** | **Effect** | **Certainty of the Evidence (GRADE)** | **Comments** |
| --- | --- | --- | --- | --- | --- |
| **Flexural Strength** | 21 | 562 | 3D-printed < milled (MD = -1.11 MPa; 95% CI [-1.29, -0.93]) and < conventional (MD = -11.93 MPa; 95% CI [-12.51, -11.36]) | **Moderate** | Downgraded for heterogeneity (variation in materials, build angles, post-curing). Risk of bias low. |
| **Surface Hardness** | 19 | 231 | 3D-printed < milled (MD = -26.49 VHN; 95% CI [-29.89, -23.10]) and < conventional (MD = -27.06 VHN; 95% CI [-29.85, -24.28]) | **Moderate** | Downgraded for inconsistency. Most studies showed lower hardness for 3D-printed materials. |
| **Fracture Toughness** | 5 | Not reported | 3D-printed < milled and conventional (e.g., 1.30 ± 0.06 vs. 4.16 ± 0.06 MPa·m½) | **Low** | Downgraded for imprecision (small sample sizes) and indirectness (lack of standard protocols). |
| **Impact Strength** | 4 | Not reported | 3D-printed < milled and conventional (e.g., 2.44 to 6.32 kJ/m² range) | **Low** | Downgraded for imprecision and heterogeneity. Few studies available. |

**Explanations of GRADE Ratings:**

- **Risk of bias:** Most studies had low risk of bias (per JBI tool).
- **Inconsistency:** Moderate to high heterogeneity in materials, printers, and protocols.
- **Indirectness:** All studies were in vitro; results may not directly translate to clinical practice.
- **Imprecision:** Some outcomes had small sample sizes or wide confidence intervals.
- **Publication bias:** Slight asymmetry in funnel plots, minimal effect after trim-and-fill.

**Tables 3S:** Table summarizing key testing conditions (e.g., specimen dimensions, loading rates, curing protocols)

| Study ID | Resin Type | Test Type | Specimen Dimensions | Sample Size | Post-Curing Protocol | Testing Machine | Loading  Rate | Standard Used (if any) |
| --- | --- | --- | --- | --- | --- | --- | --- | --- |
| Abd El-latif et al., 2021 | Vertex (Heat-PMMA), AvaDent & Polident (CAD-CAM PMMA), Harz & NextDent (3D-printed), Polyamide (Bre.Flex 2nd edition) | Flexural strength (3-point bend) | 65 × 10 × 3 mm | 10 per group | Harz: Isopropyl wash + UV cure 15 min; NextDent: UV cure 15 min; Vertex: 70°C for 9 hrs; Polyamide: 220–265°C for 15 min injection molding | Instron 3345 (UK) | 5 mm/min | ISO 20795-1:2013 |
| Abdallah et al., 2022 | Conventional PMMA, CAD/CAM PMMA, 3D-Printed Denture Base Resin | Flexural strength (3-point bending) | 64 × 10 × 3.3 mm | 10 per group | 3D-printed: post-cured with UV; others per manufacturer’s protocol | Instron universal testing machine | 5 mm/min | ISO 20795-1:2013 |
| Abdul-Monem & Hanno, 2024 | NextDent Denture 3D+, AvaDent CAD/CAM PMMA, Conventional PMMA | Flexural strength (3-point bending) | 64 × 10 × 3.3 mm | 10 per group | NextDent: UV 30 min (LC-3DPrint Box); others per manufacturer | Instron universal testing machine | 5 mm/min | ISO 20795-1:2013 |
| Adsare et al., 2024 | Avadent (CAD/CAM), Dentca & Formlabs (3D-printed), Leucitone 199 (Conventional PMMA) | Fracture toughness | 35 × 10 × 3 mm | 10 per group | 3D-printed: Not specified (UV-cured generally implied); others per lab/manufacturer protocol, not detailed | Universal Testing Machine (CSIR, India) | 1 mm/min | Not mentioned |
| Alanazi et al., 2024 | Conventional PMMA, CAD/CAM PMMA, 3D-Printed PMMA | Flexural strength (3-point bending) | 64 × 10 × 3.3 mm | 10 per group | 3D-printed: UV cured 30 min; others per manufacturer’s instructions | Instron universal testing machine | 5 mm/min | ISO 20795-1:2013 |
| Alaseef et al., 2022 | NextDent Denture 3D+, Conventional Heat-Cured PMMA | Flexural strength (3-point bending) | 64 × 10 × 3.3 mm | 10 per group | NextDent: UV cured for 30 min; Conventional: processed by heat polymerization | Universal testing machine (Instron 3345) | 5 mm/min | ISO 20795-1:2013 |
| Alshali et al., 2024 | NextDent (3D-printed), GC (Conventional heat-cured PMMA), Ivotion (CAD-CAM milled) | Flexural strength (3-point bend) | 65 × 10 × 3.3 mm | 10 per group | 3D-printed: Washed in isopropyl alcohol, UV cured for 30 min (NextDent LC-3DPrint Box) | Universal testing machine (Instron, USA) | 5 mm/min | ISO 20795-1:2013 |
| Arora et al., 2024 | NextDent Denture 3D+, Conventional PMMA | Flexural strength (3-point bending) | 65 × 10 × 3 mm | 10 per group | NextDent: UV 30 min (LC-3DPrint Box); PMMA: Water bath 100°C | Instron 3366 Universal Testing Machine | 5 mm/min | ISO 20795-1:2013 |
| Augusto et al., 2024 | VarseoSmile Denture Base (3D-printed), Probase Hot (Heat-cured PMMA) | Flexural strength (3-point bend) | 64 × 10 × 3.3 mm | 10 per group | 3D-printed: Light-cured for 20 min | Instron universal testing machine (USA) | 5 mm/min | ISO 20795-1:2013 |
| Bento et al., 2024 | Onda Cryl (Conventional & Microwave), BlueDent (Milled), SmartDent (3D-printed) | Flexural strength, Microhardness, Modulus of Elasticity | 64 × 10 × 3.3 mm | 10 per subgroup | 3D-printed: UV-cured using DLP (MoonRay Model S) printer | Universal testing machine (EMIC) | 5 mm/min | ISO 20795-1:2013, ASTM E384-11 |
| Baciu et al., 2023 | NextDent Denture 3D+, Freeprint denture, GC Temp PRINT | Flexural strength (3-point bending) | 64 × 10 × 3.3 mm | 10 per group | NextDent: LC-3DPrint Box 30 min; Freeprint: BB cure unit 30 min; GC: Labolight DUO 5 min | Lloyd LR5K Plus Universal Testing Machine | 5 mm/min | EN ISO 20795-1:2013 |
| C. Lawson et al., 2024 | NextDent (3D-printed), Avadent (Milled) | Flexural strength (3-point bend) | 64 × 10 × 3.3 mm | 10 per group | UV-cured for 30 min | Instron universal testing machine | 5 mm/min | Not mentioned |
| Cakmak et al., 2023 | Additive: NextDent Denture 3D+, FREEPRINT denture, Denturetec | Surface roughness, Vickers Hardness, Stainability | Ø10 mm × 2 mm | 10 per group | AM-N: LC-3DPrint Box 30 min at 60°C AM-F/AM-S: Otoflash G171 (4000 flashes) All cleaned with isopropanol/ethanol per protocol | Vickers Hardness Tester (M-400, Leco Corp) FRT MicroProf 100 profilometer SD Mechatronik Thermocycler for cycling | Not mentioned | Not mentioned |
| Casucci et al., 2023 | NextDent 3D+ and GC Temp Print | Flexural Strength (3-point bending) | 64 mm × 10 mm × 3.3 mm | 10 per group | 30 min in NextDent LC-3DPrint Box | Instron Universal Testing Machine | 5 mm/min | ISO 10477 |
| Di Fiore et al., 2022 | Heat-polymerized (CV), CAD-CAM milled (CAD), 3D-printed (3D) PMMA | Flexural strength (3-point bend) | 65 × 10 × 3.3 mm | 6 per group | 3D: cleaned in detergent, light-cured 20 min, stored 50 h in 37°C water | Acumen 3 (MTS Systems Corp.) | 5 mm/min | ISO 20795-1 |
| Delgado-Ruiz et al., 2024 | NextDent Denture 3D+, Freeprint denture, Cosmos Denture (3D-Printed) | Flexural strength (3-point bending) | 64 × 10 × 3.3 mm | 10 per group | Post-cured with UV light 30 min using LC-3DPrint Box | Shimadzu AGS-X Universal Testing Machine | 5 mm/min | EN ISO 20795-1:2013 |
| El Samahy et al., 2023 | Vertex (conventional PMMA), Denturetec & NextDent (3D-printed) | Flexural strength (3-point bend) | 64 × 10 × 3.3 mm | 10 per group | UV-cured for 30 min at 60°C (LC-3DPrint Box, NextDent) | Lloyd Universal Testing Machine (UK) | 5 mm/min | Not mentioned |
| Falcão et al., 2022 | Vipi Wave, NextDent 3D+, Flex Resin (3D-printed) | Flexural strength, Water sorption | 65 × 10 × 3.3 mm | 10 per group | 20 min in LC-3DPrint Box | EMIC DL-2000 testing machine | 5 mm/min | ISO 20795-1:2013 |
| HJ et al., 2024 | NextDent Denture 3D+ (3D-printed), Trevalon HI (Heat-cured) | Flexural strength (3-point bend) | 64 × 10 × 3.3 mm | 10 per group | NextDent: UV-cured for 30 min in LC-3DPrint Box | Universal Testing Machine (Instron) | 5 mm/min | Not mentioned |
| Hanno & Abdul-Monem, 2023 | Vertex (Heat-cured), 3D-printed (NextDent, Freeprint), CAD/CAM milled (IvoBase CAD) | Color stability, Surface roughness | 10 mm × 2 mm | 10 per subgroup | UV-cured for 30 min (NextDent & Freeprint); others as per manufacturer | Surface: Mitutoyo Profilometer; Color: Spectrophotometer | Not mentioned | Not mentioned |
| Gad et al., 2022 | Major.Base.20 (Heat-cured), NextDent Denture 3D+ (3D-printed) | Flexural strength, Impact strength, Hardness, Surface roughness | 64×10×3.3 mm (flexural), 50×6×4 mm V-notch (impact), 10×2 mm (others) | 10 per group per test (total 120) | UV cure 10 min in glycerol bath (per manufacturer’s protocol) | Instron E3000, Charpy tester, Vickers tester, Contour Gt-K1 profilometer | 5 mm/min (flexural) | ISO 20795-1:2013 |
| Li et al., 2023 | NextDent Denture 3D+ (3D-printed), PMMA (conventional) | Flexural strength (3-point bend) | 64 × 10 × 3.3 mm | 10 per group | Washed with alcohol, UV cured for 30 min at 60°C (NextDent LC-3DPrint Box) | Universal Testing Machine (Instron) | 5 mm/min | Not mentioned |
| Li et al., 2024 | NextDent 3D+, DentaBASE (3D-printed) | Flexural strength, Surface hardness | 64 × 10 × 3.3 mm (flexural), 10 × 2 mm (hardness) | 10 per group | UV cured for 30 min at 60°C (LC-3DPrint Box) | Instron universal testing machine | 5 mm/min | ISO 20795-1:2013 |
| J et al., 2024 | NextDent Denture 3D+, Trevalon HI (heat-cured PMMA) | Flexural strength (3-point bend) | 64 × 10 × 3.3 mm | 10 per group | Washed with alcohol, UV cured for 30 min (NextDent LC-3DPrint Box) | Universal Testing Machine (Instron) | 5 mm/min | Not mentioned |
| M. Fouda et al., 2022 | Heat-polymerized (HP), AvaDent, IvoCad, ASIGA, FormLabs, NextDent | Flexural strength, Elastic modulus, Surface hardness | 64×10×3.3 mm (flexural); 15×10×2.5 mm (hardness) | 10 per test per material | 3D-printed: Washed with isopropanol; others: not mentioned | Instron Model 8871 | Not mentioned | ISO 20795-1:2013 |
| M. Fouda et al., 2023 | NextDent, IvoBase CAD, AvaDent | Flexural strength, Surface roughness | 64×10×3.3 mm; 15×10×2.5 mm (roughness) | 10 per group | Cleaned with isopropanol, UV-cured 30 min at 60°C | Instron Model 8871 | Not mentioned | ISO 20795-1:2013 |
| M et al., 2021 | Heat-polymerized (PMMA), NextDent Denture 3D+ | Flexural strength (3-point bend) | 64 × 10 × 3.3 mm | 10 per group | UV cure 30 min at 60°C in LC-3DPrint Box | Instron universal testing machine | 5 mm/min | ISO 20795-1:2013 |
| Moussa et al., 2024 | NextDent 3D+, Trevalon HI (heat-cured PMMA) | Flexural strength (3-point bend) | 64 × 10 × 3.3 mm | 10 per group | UV-cured in NextDent LC-3DPrint Box for 30 min at 60°C | Instron universal testing machine | 5 mm/min | Not mentioned |
| N. Al-Dwairi et al., 2022 | 3D-printed (NextDent 3D+), heat-polymerized (Trevalon) | Flexural strength (3-point bend) | 64 × 10 × 3.3 mm | 10 per group | Washed with alcohol and UV cured for 30 min | Instron universal testing machine | 5 mm/min | Not mentioned |
| MA et al., 2022 | 3D-printed (NextDent, Asiga), conventional PMMA | Flexural strength, Hardness, Roughness | 64 × 10 × 3.3 mm (flexural); 10 × 2 mm (others) | 10 per group | UV cure for 30 min after washing | Instron machine, Vickers tester, Profilometer | 5 mm/min | ISO 20795-1:2013 |
| NA, 2024 | ASIGA DentaBase (3D-printed), IvoBase CAD (milled) | Flexural Strength, Surface Roughness | 65 × 10 × 3.3 mm | 22 per group (3 groups, total 66) | Washed with isopropyl alcohol, cured 20 min in Asiga Flash chamber | Lloyds Instruments Model LRX | 1 mm/min | ISO standard (number not specified) |
| Neves et al., 2022 | 3D-printed (NextDent 3D+), Heat-polymerized PMMA (control) | Flexural strength (3-point bend), Color stability | 64 × 10 × 3.3 mm | 10 per group | UV cure 30 min using LC-3DPrint Box | Instron universal testing machine | 5 mm/min | Not mentioned |
| O et al., 2024 | NextDent 3D+, Trevalon HI (heat-cured PMMA) | Flexural strength (3-point bend) | 64 × 10 × 3.3 mm | 10 per group | UV cure 30 min at 60°C using LC-3DPrint Box | Instron universal testing machine | 5 mm/min | Not mentioned |
| Temizci & Bozogullari, 2024 | NextDent 3D+, Heat-cured PMMA | Flexural strength (3-point bending) | 64 × 10 × 3.3 mm | 10 per group | UV cure 30 min using NextDent LC-3DPrint Box | Instron universal testing machine | 5 mm/min | ISO 20795-1:2013 |
| V et al., 2020 | 3D-printed resin (Asiga DentaBASE), conventional PMMA | Flexural strength (3-point bending) | 64 × 10 × 3.3 mm | 10 per group | Cured for 30 min in Asiga Flash post-curing unit | Instron universal testing machine | 5 mm/min | ISO 20795-1:2013 |
| Souza et al., 2024 | Dental SG Resin (Formlabs), Freeprint Temp, E-Dent 400 (3D-Printed) | Flexural strength (3-point bending) | 25 × 2 × 2 mm | 10 per group | Post-cured with UV light 20 min using Form Cure device | EMIC DL 10000 | 0.5 mm/min | ISO 10477 |
| Z et al., 2024 | Procryla (Conventional PMMA), Yamahachi (Subtractive CAD/CAM), Curo Denture (Additive 3D-Printed) | Shear bond strength (SBS) | 7.5 mm diameter × 2 mm | 10 per group (300 total) | Procryla: 90 min @ 74°C + 30 min @ 100°C; Yamahachi: CAD/CAM milled; Curo: UV 3 min @ 60°C | Lloyd Universal Testing Machine | 1 mm/min | Not mentioned |
| Zahel et al., 2024 | NextDent Denture 3D+, Paladon 65 (heat-cured PMMA) | Flexural strength (3-point bending) | 64 × 10 × 3.3 mm | 10 per group | UV cure 30 min using LC-3DPrint Box (NextDent) | Zwick/Roell Z005 machine | 5 mm/min | ISO 20795-1:2013 |

1 Choi, J. J. E. *et al.* Adhesion of Denture Characterizing Composites to Heat-Cured, CAD/CAM and 3D Printed Denture Base Resins. *J Prosthodont* **30**, 83-90, doi:10.1111/jopr.13291 (2021).

2 Çakmak, G. *et al.* Effect of coffee thermal cycling on the surface properties and stainability of additively manufactured denture base resins in different layer thicknesses. *J Prosthodont* **34**, 157-166, doi:10.1111/jopr.13803 (2025).

3 Takhtdar, M., Azizimoghadam, N., Kalantari, M. H. & Mohaghegh, M. Effect of denture cleansers on color stability and surface roughness of denture bases fabricated from three different techniques: Conventional heat-polymerizing, CAD/CAM additive, and CAD/CAM subtractive manufacturing. *Clin Exp Dent Res* **9**, 840-850, doi:10.1002/cre2.763 (2023).

4 Alzaid, M. *et al.* The Effect of Salivary pH on the Flexural Strength and Surface Properties of CAD/CAM Denture Base Materials. *Eur J Dent* **17**, 234-241, doi:10.1055/s-0042-1749160 (2023).

5 Grachev, D. I. *et al.* Ranking Technologies of Additive Manufacturing of Removable Complete Dentures by the Results of Their Mechanical Testing. *Dentistry Journal* **11**, 265 (2023).

6 Janyaprasert, P., Kamonkhantikul, K., Homsiang, W. & Arksornnukit, M. Effect of thermocycling on tensile bond strength of autopolymerized, heat-polymerized, milled, and 3D printed denture base materials bonded to 4 different denture liners: an in vitro study. *BMC Oral Health* **24**, 1000 (2024).

7 Jiang, C. P., Hentihu, M. F. R., Lee, S. Y. & Lin, R. Multiresin Additive Manufacturing Process for Printing a Complete Denture and an Analysis of Accuracy. *3D Print Addit Manuf* **9**, 511-519, doi:10.1089/3dp.2021.0007 (2022).

8 Ravalec, G. *et al.* A comparison of conventional and machinable PMMA materials for denture. *Computer Methods in Biomechanics and Biomedical Engineering* **23**, S253-S254, doi:10.1080/10255842.2020.1815325 (2020).

9 Mann, R. S. & Ruse, N. D. Fracture toughness of conventional, milled and 3D printed denture bases. *Dental Materials* **38**, 1443-1451, doi:<https://doi.org/10.1016/j.dental.2022.06.029> (2022).

10 Tzeng, J.-J., Yang, T.-S., Lee, W.-F., Chen, H. & Chang, H.-M. Mechanical Properties and Biocompatibility of Urethane Acrylate-Based 3D-Printed Denture Base Resin. *Polymers* **13**, 822 (2021).
